# Development of cytosine and adenine base editors for maize precision breeding

**DOI:** 10.1111/jipb.13964

**Published:** 2025-07-11

**Authors:** Xiao Fu, Nan Wang, Lina Li, Dexin Qiao, Xiantao Qi, Changlin Liu, Zhaoxu Gao, Chuanxiao Xie, Jinjie Zhu

**Affiliations:** ^1^ State Key Laboratory of Crop Gene Resources and Breeding, Institute of Crop Sciences Chinese Academy of Agricultural Sciences Beijing 100081 China; ^2^ National Nanfan Research Institute Chinese Academy of Agricultural Sciences Sanya 572025 China

**Keywords:** adenine BE, cytosine BE, herbicide resistance, multiplex editing, *Zea mays*, *ZmACC*

## Abstract

Base editing technologies can improve crops, but their efficiency in maize remains suboptimal. This study attempts to overcome these limitations by examining optimized cytosine and adenine base editors (CBEs and ABEs), namely evoAPOBEC1, evoFERNY, evoCDA1, TadA8.20, and TadA8e, for precise genome editing in transient and stable expression maize cells. Employing a seed fluorescence reporter (SFR) system for rapid screening of BE transformants and transgene‐free progenies, we enhanced editing efficiencies and heritability. Notably, TadA8.20 and evoCDA1 attained multiplexed editing efficiencies of up to 100.0% and 79.0% at the tested loci, respectively, with some homozygous and bi‐allelic mutants exceeding 72.4% and 73.7%. Precise editing of *ZmACC1*/*2* (acetyl‐CoA carboxylase) improved herbicide resistance, with *ZmACC2* mutants displaying improved performance. This study advances crop genetic engineering by facilitating robust, multi‐locus modifications without altered agronomic performance, enhancing herbicide tolerance in maize. The successful utilization of these BE is a significant step forward in agricultural biotechnology and precision breeding.

## Graphical abstract

A series of maize cytosine and adenine base editors was developed using various deaminase variants, enabling efficient multiplex genome editing in maize. Precise editing of the *ZmACC1/2* genes generated herbicide‐tolerant maize germplasm, offering a solution for weed management in maize‐soybean intercropping systems.

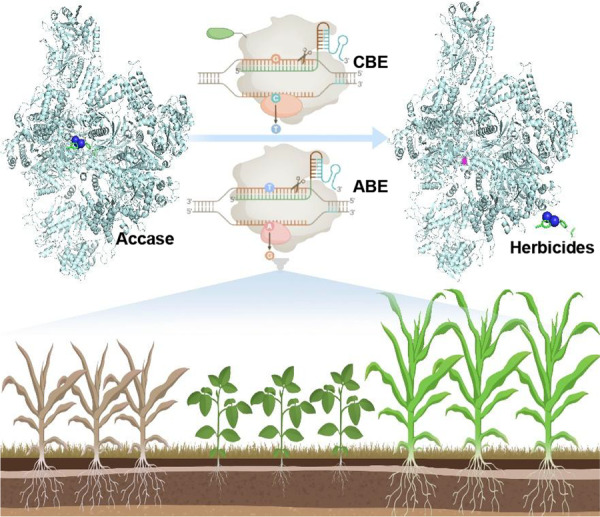

## INTRODUCTION

Genetic variation is foundational for crop enhancement and breeding, with single‐nucleotide or oligonucleotide polymorphisms (SNPs/ONPs) acting as prevalent contributors to genetic diversity (Wright et al., 2005). Maize, a significant food crop, obtains genetic and phenotypic diversity from SNPs (Li et al., 2013; Sun et al., 2018; Wang et al., 2023). Identifying and utilizing key SNPs associated with favorable traits is crucial for improving crop performance (Xing et al., 2015). Recent advancements in genome editing, including base editors (BE) and prime editing (PE), provide direct and efficient approaches to induce targeted mutations, accelerating genetic enhancements (Li et al., 2018; Hua et al., 2018; Lin et al., 2020).

Base editor systems employ fusion proteins that integrate catalytically impaired Cas nuclease with DNA deaminases or glycosylases, allowing base transition or transversion mutations without double‐strand breaks. Diverse deaminases have been used in the development of BEs like cytosine base editors (CBEs) (Komor et al., 2016), adenine base editors (ABEs) (Gaudelli et al., 2017), dual base editors (Xu et al., 2021a, 2021b), CGBE (Zeng et al., 2022), AYBE (Zeng et al., 2022; Tong et al., 2023), AKBE (Wu et al., 2023), as well as TBE (Tong et al., 2024; Ye et al., 2024). Highly efficient CBE deaminases, including evoAPOBEC1, evoFERNY, and evoCDA1, with ABE deaminases like TadA8.20 and TadA8e, have been refined via enzyme evolution (Thuronyi et al., 2019; Gaudelli et al., 2020; Richter et al., 2020). It is noteworthy that the re‐engineering of the adenine deaminase TadA also enables efficient cytosine editing (Chen et al., 2023; Neugebauer et al., 2023). These optimized CBEs and ABEs exert promising results in rice (Zeng et al., 2020; Yan et al., 2021a; Fan et al., 2024). PE technology, which integrates Cas9 with reverse transcriptase and pegRNA, enables precise genome editing, allowing for 12 types of base substitution integration (Anzalone et al., 2019). However, BE and PE technologies in maize are still in their infancy and require further optimization. Previously, rAPOBEC1 deaminase use in maize attained target editing efficiencies of 13.9% in stable T1 transformants (Li et al., 2020). Recently, the TadA8e‐based AYBE approach produced a mutation efficiency of nearly 25.2%, accompanied by a chimerism rate of 10% (Zhong et al., 2024). Consequently, developing highly efficient BE editors is essential for precision breeding in maize and genetic improvement.

The robustness of genome editing in crops like maize can be significantly improved by integrating reporter systems, which is crucial for identifying transgene‐positive and transgene‐negative individuals in a time‐effective and cost‐effective manner (Gao, 2021). Our previous work produced a dual fluorescence protein system using embryo‐specific green fluorescent protein and endosperm‐specific red fluorescent protein expression, useful in maize haploids (Dong et al., 2018), maintainer lines (Qi et al., 2020), and seed fluorescence reporters (SFRs) (Yan et al., 2021b). Moreover, the RUBY reporter, requiring no specialized equipment, has been used to monitor transformation events across diverse crops (He et al., 2020). These reporter systems enable the rapid identification and selection of edited plants, improving the isolation of individuals lacking foreign genetic material.

In this study, we evaluated four CBEs—evoAPOBEC1, evoFERNY, evoCDA1, and RrA3F (Yu et al., 2020)—and two ABEs—TadA8.20 and TadA8e—in maize transient and stable expression cells. We explored multiplex editing capacities and efficiencies using SFR methodologies. By triggering targeted base edits in *ZmACC1* and *ZmACC2* genes, we generated a series of single and double mutants exhibiting herbicide resistance. Our findings provide robust CBE and ABE tools for maize, thereby contributing to the development of herbicide‐resistant maize germplasm and agricultural biotechnology.

## RESULTS

### Testing current BE in the maize transient expression assays

**Figure 1:**
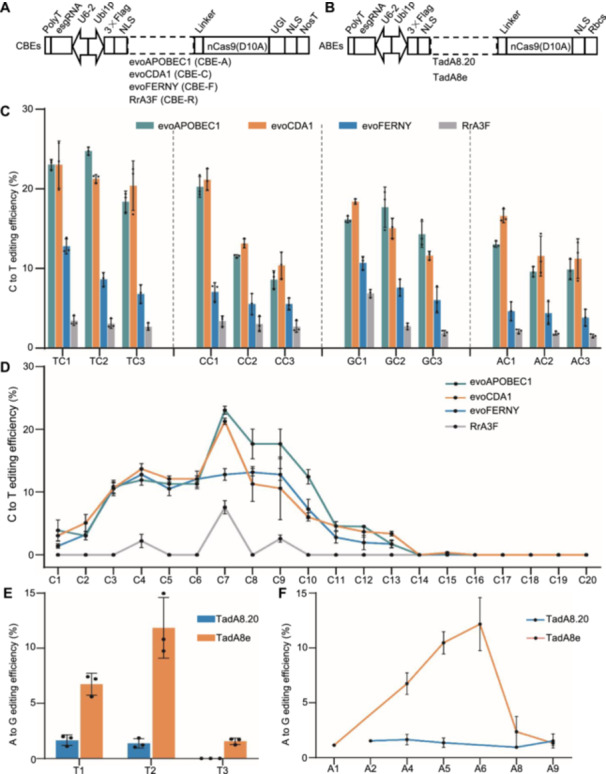
Editing efficiency and windows of CBEs and ABEs in endogenous site protoplasts of maize **(A)** Cytosine base editor systems using evoAPOBEC1, evoCDA1, evoFERNY, and RrA3F, respectively. **(B)** Adenine base editor systems using TadA8e or TadA8.20. **(C–F)** Editing efficiency and windows of various CBEs at endogenous loci in maize. **(E**, **F)** Editing efficiency and window profiles of various ABEs at endogenous loci in maize.

To produce efficient CBEs and ABEs, we examined deaminase variants. Cytosine deaminases, including evoAPOBEC1 (CBE‐A), evoCDA1 (CBE‐C), evoFERNY (CBE‐F), and the variant RrA3F (CBE‐R) were examined (Figure 1A). Adenine deaminases encompassed TadA8e (ABE‐TadA8e) and TadA8.20 (ABE‐TadA8.20) (Figure 1B). To improve their functionality, these deaminases contained a bipartite nuclear localization signal (NLS) alongside a 32‐amino acid linker on either end of their expression cassettes. After codon optimization and synthesis, these elements were appended to the N‐terminus of Cas9 (D10A), with the C‐terminus of the CBEs further fused to a uracil glycosylase inhibitor (UGI). Target sites for CBEs and ABEs were designed, employing an upgraded sgRNA backbone, driven by the maize endogenous *ZmU6‐2* promoter.

To examine CBEs, we designed three target sites for each context motif TC/CC/GC/AC (Table S1). We evaluated the editing efficiency, editing window, and context dependence of the CBEs in maize protoplasts via transient transformation. Both evoCDA1 and evoAPOBEC1 had similar and significantly higher editing efficiencies than evoFERNY and RrA3F, with RrA3F exhibiting the lowest efficiency (Figure 1C). Analysis of the target sequence context of the four deaminases revealed a sequence preference, with evoAPOBEC1 and evoCDA1 exhibiting preferential targeting of TC motifs, evoFERNY having the lowest editing activity at the AC site, and RrA3F exhibiting non‐preferential editing (Figure 1C). The base editing window was examined at various positions across the 12 target sites' C‐editing efficiency. The editing window for evoAPOBEC1, evoCDA1, and evoFERNY remained consistent from C1 to C14, with optimal editing activity at position C7 for evoAPOBEC1 and evoCDA1. Conversely, RrA3F displayed a narrower editing window, with activity limited to C4, C7, and C9 (Figure 1D).

For ABEs, we chose three target sites to assess the editing efficiency of TadA8e and TadA8.20 base editors (Table S1). The base editing efficiency of TadA8e ranged from 1.24% to 11.28%, while that of TadA8.20 ranged from 0.88% to 1.85% (Figure 1E). TadA8e exhibited increased editing activity compared to TadA8.20 in maize protoplasts. The base editing window for ABE‐8e spanned A1–A9, whereas ABE‐8.20 covered A2–A9. Peak editing activity was identified at positions A5–A7 (Figure 1F).

We evaluated the editing efficiencies of various base editors in stable transgenic lines. Targeting sites for CBEs and ABEs were designed at herbicide‐resistance‐associated loci in *ZmACC* genes (Liu et al., 2020; Wang et al., 2022), with *ZmACC1* and *ZmACC2* targeted simultaneously. Transient protoplast expression assays uncovered that all CBE systems mediated base editing at *ZmACC1/2*, with editing efficiencies of evoCDA1 > evoAPOBEC1 > evoFERNY > RrA3F (Figure S1A, C). Notably, evoCDA1, evoAPOBEC1, and evoFERNY had similar site profiles, while RrA3F was restricted to C7 and C8 positions (Figure S1B, D). For ABE‐targeted sites, TadA8e‐ABE exhibited significantly increased base editing efficiency compared with TadA8.20‐ABE (Figure S1E, G), with peak activity at A5 and A6 positions (Figure S1F, H).

### Base editor robustness characterized using SFRs

**Figure 2:**
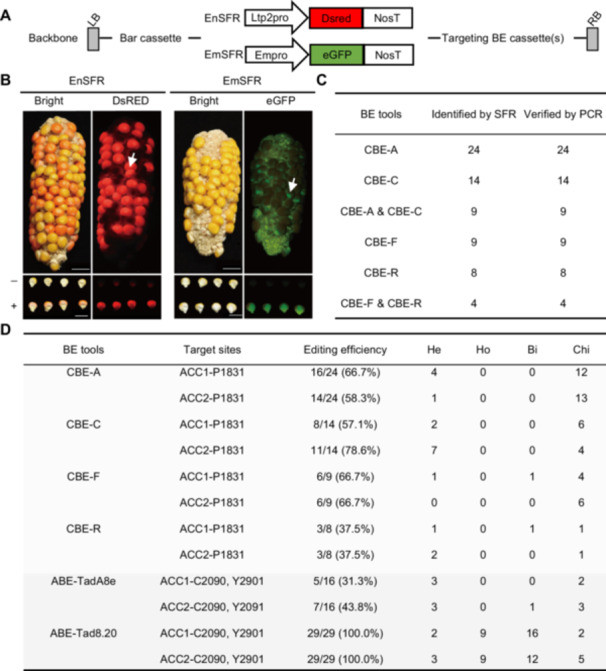
Tested BEs and transformant screening using SFR **(A)** Vector backbone. LB, T‐DNA left border; Bar, *BlpR* gene; EnSFR, endosperm seed fluorescence reporter /EmSFR, embryo seed fluorescence reporter; different CRISPR‐Cas9 base editing cassette; RB, T‐DNA right border. **(B)** Visible sorting of seeds with BE editing machinery mutants using EnSFR or EmSFR. EnSFR‐DsRED‐fluorescence was obtained under 550 nm excitation, and EmSFR‐eGFP‐fluorescence was obtained at 480 nm excitation. Scale bars, 2 cm. **(C)** T0 positive transformants were verified using SFR and polymerase chain reaction (PCR). **(D)** Editing efficiency and genotype of cytosine/adenine base editors (CBE/ABE) editors on *ZmACC* targets in T0 transgenic lines. Bi, bi‐allelic mutation; Chi, chimeric mutation; He, heterozygous mutation; Ho, homozygous mutation.

To enable rapid screening, we integrated our SFR system for maize (Dong et al., 2018) into the new CBEs. Specifically, we added a red fluorescent protein expression cassette targeting the endosperm (EnSFR) and a green fluorescent protein expression cassette targeting the embryo (EmSFR) into diverse CBE constructs (Figures 2A, S2), allowing the screening of different transformation vectors (Figure 2B). Maize transformation was carried out using *Agrobacterium*‐mediated delivery. In total, 47 T0 transgenic events were obtained for the combined CBE‐A&CBE‐C transformation and 21 T0 lines for the mixed CBE‐F&CBE‐R transformation.

Using handheld fluorescence lamps with excitation wavelengths of 480 and 550 nm, seeds were sorted according to fluorescence. Seeds expressing DsRED under the control of an endosperm‐specific promoter produced red fluorescence at 550 nm. In contrast, those with an embryo‐specific promoter driving eGFP expression produced green fluorescence at 480 nm (Figure 2B). We identified 24 transformants carrying CBE‐A, 14 carrying CBE‐C, and nine carrying CBE‐A and CBE‐C. Additionally, we identified nine transformants carrying CBE‐F, eight carrying CBE‐R, and four carrying CBE‐F and CBE‐R (Figure 2C). These results were verified using molecular markers specific for different cytosine deaminases, aligning with fluorescence‐based identification (Figure S3). This integration of the SFR system improves the robustness and efficiency of screening in developing and applying BEs.

### Base editor editing efficiencies in stable expression lines

To assess the editing efficiencies of CBEs and ABEs, we amplified T0 transgenic plant target sites and assessed the editing efficiency using Hi‐TOM (Liu et al., 2019). Our data uncovered that the C‐to‐T base editing efficiencies of CBE‐A, CBE‐C, CBE‐F, and CBE‐R at two loci ranged from 58.3% to 66.7%, 57.1% to 78.6%, 66.7%, and 37.5% (Figure 2D). The evoCDA1 variant exhibited the highest editing efficiency, followed closely by evoFERNY and evoAPOBEC1, whereas RrA3F had the lowest efficiency. We classified the genotypes of the target sites into chimeric (mutant reads < 30%), heterozygous (30% < mutant reads < 70%), and homozygous or bi‐allelic (mutant reads > 70%). CBE‐F and CBE‐R mediated one bi‐allelic genotype, and many CBE‐evoCDA1‐mediated plants were heterozygous (Figure 2D).

We also assessed the A‐to‐G editing activity of ABE variants. Adenine base editor‐TadA8e exhibited editing efficiencies of 31.3% and 43.8% at two endogenous target sites, while ABE‐TadA8.20 achieved efficiencies of 100.0% at both sites. Adenine base editor‐TadA8e mediated one bi‐allelic genotype, with over half classified as heterozygous, whereas over 72.4% of ABE‐TadA8.20‐mediated plants were homozygous or bi‐allelic. These findings offer a comprehensive overview of the editing efficiencies for BEs in stable expression lines, underscoring the potential of ABE‐TadA8.20 and CBE‐evoCDA1 in maize genome editing.

### Efficiency optimization and multiplex capacity of CBE‐C

**Figure 3:**
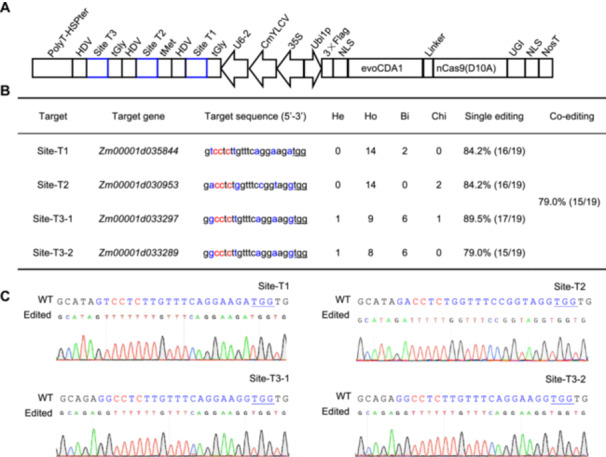
Efficiency optimization and multiplex capability of CBE‐C **(A)** CBE‐C vector for efficiency optimization and multiplex capacity. **(B)** The editing efficiency for evoCDA1‐based CBE‐C at four maize endogenous targets. The PAM sequence is underlined. Bi, bi‐allelic mutation; Chi, chimeric mutation; He, heterozygous mutation; Ho, homozygous mutation. **(C)** Representative Sanger sequencing chromatograms of targets from a quadruplex homozygous mutant in T_0_ generation. Target sequences are denoted in blue, the PAM sequence is underlined, and targeted base mutations are highlighted in red.

To improve the editing performance of CBE‐C and assess its multiplexing potential, we developed a tandem sgRNA expression that integrated a chimeric *35S‐CmYLCV‐ZmU6* promoter and tRNA and HDV ribozyme processing elements (Figure 3A). This system targeted four maize homologs of the *Arabidopsis DA1* gene. Specifically, Target 1 targets *Zm00001d035844*, Target 2 targets *Zm00001d030953*, and Target 3 targets both *Zm00001d033297* (Site‐T3‐1) and *Zm00001d033289* (Site‐T3‐2). Via stable transformation, we obtained 19 T0 transgenic plants. Hi‐TOM data analysis revealed editing efficiencies of 84.2%, 84.2%, 89.5%, and 79.0% for the four targets, respectively. Most T0 lines (15 out of 19, 79.0%) had simultaneous edits across all genes. Genotypic analysis demonstrated high frequencies of homozygous or bi‐allelic genotypes, with rates of 100%, 87.5%, 88.2%, and 93.3% for each target (Figure 3B). Notably, 73.7% of the edited plants (14 out of 19) had homozygous or bi‐allelic genotypes across all four targets, indicating a pronounced editing efficiency in stable transgenic plants (Figure 3C; Table S2). These findings suggest that using the *35S‐CmYLCV‐ZmU6* promoter with CBE‐C effectively achieves highly efficient, homologous, and multiplex editing.

### Mutations of *ZmACC1*/*2* edits are heritable

**Figure 4:**
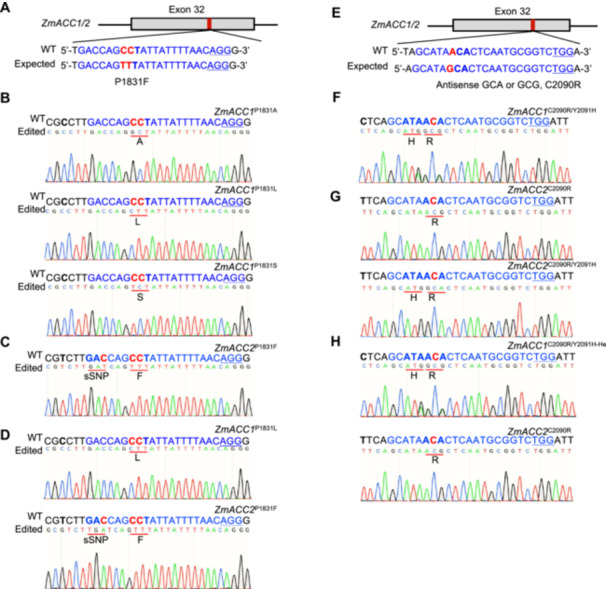
Generation of homologous target edits of *ZmACC1*/*2* at the T2 generation **(A)** Cytosine base editors (CBEs) targeting the P1831 coding sequence of *ZmACC1*/*2*. **(B–D)** Sanger sequencing chromatograms of homozygous mutations in ZmACC1^P1831A^, ZmACC1^P1831L^, and ZmACC1^P1831S^ **(B)**, ZmACC2^P1831F^ **(C)**, and ZmACC1^P1831L^ZmACC2^P1831F^ **(D)**. **(E)** Adenine base editor (ABE) targeting the C2090 coding sequence of *ZmACC1*/*2*. **(F–H)** Sequencing chromatograms from homozygous mutations of ZmACC1^C2090R/Y2091H^ **(F)**, ZmACC2^C2090R^ and ZmACC2^C2090R/Y2091H^ **(G)**, and ZmACC1^C2090R/Y2091H‐He^ZmACC2^C2090R^ **(H)**. In panels **(A–H)**, target sequences are denoted in blue, the PAM sequences are underlined, and codons for Q1830, P1831, C2090, and Y2091 of *ZmACC1*/*2* are bolded. Key base mutations are identified in red. sSNP, synonymous mutation.

In this study, CBEs were engineered to target the P1831 coding sequence in *ZmACC1*/*2*, enabling specific P1831 missense mutations via directed C7 or C8 alterations (Figure 4A). Adenine base editors were designed to edit the C2090 coding sequence of *ZmACC1*/*2*, enabling C2090 missense mutations through A6 and A8 substitutions (Figure 4E). From T0 to T2 generations, we combined SFR and polymerase chain reaction (PCR) screening to obtain T2 progenies harboring homologous mutation and “transgene‐free” (Table S3). We successfully derived three homozygous mutants for ZmACC1, specifically ZmACC1^P1831A^, ZmACC1^P1831L^, and ZmACC1^P1831S^ (Figure 4B), alongside a ZmACC2^P1831F^ homozygous mutant (Figure 4C) and a dual‐gene homozygous line containing ZmACC1^P1831L^ and ZmACC2^P1831F^ (Figure 4D). For ABEs‐induced mutants, homozygosity was achieved for ZmACC1^C2090R/Y2091H^ (Figure 4F) alongside ZmACC2^C2090R^ and ZmACC2^C2090R/Y2091H^ (Figure 4G). Moreover, we isolated a dual‐gene mutated line with ZmACC1^C2090R/Y2091H‐He^ ZmACC2^C2090R^ (Figure 4H). Notably, most of the *ZmACC1*/*2* edits can be homozygous, whereas only heterozygous mutants of ZmACC1^C2090R/Y2091H^ZmACC2^C2090R^ could be obtained. This suggests a possible association with embryonic lethality resulting from concurrent homozygosity at both loci.

### Herbicide resistance and field performance of *ZmACC1*/*2* edited mutants

**Figure 5:**
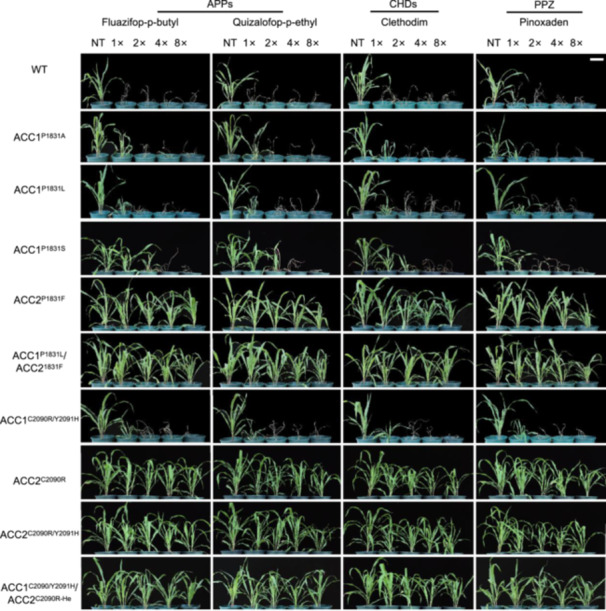
Herbicide response in heritable mutants at the T3 generation Phenotypes of nine *ZmACC1* and *ZmACC2* mutant lines (ZmACC1^P1831A^, ZmACC1^P1831L^, ZmACC1^P1831S^, ZmACC2^P1831F^, ZmACC1^P1831L^ZmACC2^P1831F^, ZmACC1^C2090R/Y2091H^, ZmACC2^C2090R^, ZmACC2^C2090R/Y2091H^, and ZmACC1^C2090R/Y2091H‐He^ZmACC2^C2090R^) and the wild‐type, treated using four representative ACCase‐inhibiting herbicides. Treatments were administered at the two‐ to three‐leaf stage with fluazifop‐p‐butyl, quizalofop‐p‐ethyl, clethodim, and pinoxaden at 1×, 2×, 4×, and 8× the minimum lethal concentrations. The minimum lethal concentrations for these herbicides are: fluazifop‐p‐butyl (13.0 g a.i./ha), quizalofop‐p‐ethyl (12.6 g a.i./ha), clethodim (12.2 g a.i./ha), and pinoxaden (13.5 g a.i./ha). Phenotypes were documented 16 d after treatment, employing the same NT control for comparison across concentrations. Scale bar, 10 cm. NT, no treatment.

To evaluate the herbicide tolerance of these mutants, “transgene‐free” T3 generation plants at the two‐ to three‐leaf stage were treated with four representative herbicides, each representing ACCase inhibitors: aryloxyphenoxypropionates (APPs), cyclohexanediones (CHDs), and phenylpyrazolines (DENs). The mutants survived treatments with 13.0 g a.i./ha of fluazifop‐butyl, 12.6 g a.i./ha of quizalofop‐p‐ethyl, 12.2 g a.i./ha of clethodim, and 13.5 g a.i./ha of pinoxaden, while the wild‐type (WT) plants died. *ZmACC1*/2 mutants had various levels of tolerance. ZmACC1^P1831A^, ZmACC1^P1831L^, and ZmACC1^C2090R/Y2091H^ survived the minimal (1×) concentration of all four herbicides, whereas ZmACC1^P1831S^ tolerated nearly 2× concentrations of fluazifop‐butyl, quizalofop‐p‐ethyl, and clethodim. All surviving *ZmACC1* mutants experienced significant growth limitations compared to untreated controls. Homozygous mutants ZmACC2^P1831F^, ZmACC2^C2090R^, and ZmACC2^C2090R/Y2091H^ exhibited robust tolerance at all herbicide concentrations, with growth rates mirroring untreated controls and only slight developmental delays at 8× concentrations of haloxyfop‐P‐methyl and pinoxaden. Double‐gene mutants like ZmACC1^P1831L^ZmACC2^P1831F^ and ZmACC1^C2090R/Y2091H‐He^ZmACC2^C2090R^ exhibited performances similar to *ZmACC2* mutants under herbicide treatments (Figure 5). These findings suggest that *ZmACC2* confers resistance to ACCase‐inhibiting herbicides.

**Figure 6:**
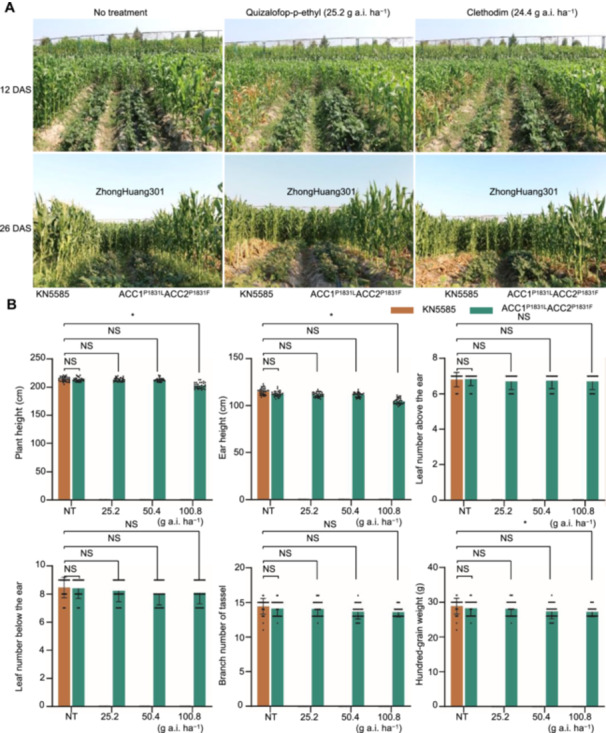
Field phenotyping of ZmACC1^P1831L^ZmACC2^P1831F^ homozygous mutants in an intercropping system with soybean under quizalofop and clethodim treatments **(A)** The performance of wild‐type and mutant plants subjected to 25.2 g a.i./ha of quizalofop‐p‐ethyl and 24.4 g a.i./ha clethodim at the four‐ to five‐leaf stage. Phenotypic data were obtained at 12 and 26 d post‐herbicide application. **(B)** The agronomic traits of the ZmACC1^P1831L^ZmACC2^P1831F^ mutant with or without quizalofop‐p‐ethyl treatment. The plant height, ear height, number of leaves above the ear, number of leaves below the ear, number of tassel branches, and 100‐grain weight were collected for significance analysis (*P* < 0.05). Values represent means ± standard deviation (*n* = 30). NS, no significance.

Field evaluations of *ZmACC1*/*2* edited mutants and wild‐type plants uncovered further insights into herbicide tolerance. Double‐gene mutants ZmACC1^P1831L^ZmACC2^P1831F^ and WT were intercropped with soybeans (ZhongHuang301) and administered quizalofop and clethodim at 2×, 4×, and 8× concentrations during the four‐ to five‐leaf stages. At 12 d after spraying (DAS), wild‐type plants exhibited wilting of the upper leaves and chlorosis, while mutant plants exhibited no visible growth impairments. By 26 DAS, all wild‐type maize and surrounding weeds had died, while mutant plants remained unaffected under both herbicide treatments (Figure 6A). Agronomic traits like plant height, ear height, leaf count (above and below the ear), tassel branch number, and 100‐seed weight were recorded for mutant and wild‐type plants under herbicide and control treatments. There were no significant differences in these traits between untreated mutant and wild‐type controls (Figure 6B). In herbicide‐treated conditions, wild‐type plants perished regardless of concentration. Conversely, mutant plants retained agronomic traits comparable to untreated controls under 2× and 4× herbicide concentrations, with only slight reductions in plant height, ear height, and 100‐seed weight under 8× herbicide treatment (Figures S5, S6). These findings suggest that maize ACCase mutants produced by base editing can effectively serve in field weed management, offering a practical solution for improving herbicide tolerance.

## DISCUSSION

In this study, we comprehensively evaluated CBEs and ABEs in both transient and stable expression systems in maize, highlighting significant advancements in BE tools for precision crop breeding. Among the phage‐evolved cytosine deaminases tested, evoAPOBEC1, evoFERNY, and evoCDA1 exhibited markedly enhanced editing efficiencies. The editing performance followed: evoCDA1 > evoAPOBEC1 > evoFERNY > RrA3F, with evoCDA1 achieving high coediting efficiencies at multiple loci, predominantly generating homozygous mutations. This level of efficiency exceeds that reported for PE technologies in maize (Jiang et al., 2020; Qiao et al., 2023), highlighting optimized evoCDA1 as a versatile tool for multi‐locus and stable genome modifications in crop improvement. However, context‐dependent editing preferences were also observed with different base editors. Specifically, evoAPOBEC1, evoFERNY, and evoCDA1 exhibited a strong preference for editing “TC” motifs, which is consistent with previous findings (Komor et al., 2016; Xu et al., 2025). To address this limitation, recent studies have employed structure‐guided approaches to identify highly efficient cytidine deaminases with reduced sequence‐context dependence (Xu et al., 2025). In our transient expression assay, all loci investigated with ABE‐TadA8e had significantly higher editing activity than ABE‐TadA8.20. However, in stable transformation lines, the editing efficiency and homozygous mutant proportion obtained with ABE‐TadA8.20 were significantly higher than those obtained with ABE‐TadA8e. This discrepancy may be partially attributed to the 6‐month interval between the transformation experiments. Supporting this, our AKBE tools, recently developed using the two TadA variants, showed that the editing efficiency at this locus was consistently higher with the ABE‐TadA8e‐based construct (data not shown). In addition, re‐engineering of adenine deaminases has enabled efficient cytosine editing, offering a smaller molecular size, reduced off‐target activity, and a simplified architecture for dual base editors (Neugebauer et al., 2023). With the continued development of more efficient and functionally complementary precision base editing tools, the construction of saturated gene mutation libraries will become increasingly straightforward.

A major challenge in applying BE tools, particularly in clinical therapies, is the occurrence of off‐target effects. Off‐target effects can occur at both the DNA and RNA levels (Jin et al., 2019; Zhou et al., 2019). DNA‐level off‐targets can be categorized into sgRNA‐dependent and sgRNA‐independent off‐targets. sgRNA‐dependent off‐targets arise from the specificity of the sgRNA (Doench et al., 2016). These sites can be predicted using off‐target prediction tools and confirmed by molecular detection. In this study, genotypic analysis of potential off‐targets in high‐editing‐efficiency transformed plants did not uncover any off‐target events (Table S4). In contrast, sgRNA‐independent DNA and RNA off‐targets are often attributed to the nonspecific deamination activity of the deaminase on ssDNA or ssRNA (Yang et al., 2017; McCann et al., 2023). We utilized the high‐fidelity TadA‐V106W variant, with diverse base editors based on this variant showing no significant off‐target activity in plants (Fan et al., 2024). Furthermore, RNA‐level off‐targets did not result in stable transmission to progeny; therefore, RNA‐level off‐targets do not pose a critical issue in BE plant applications.

Using CBEs and ABEs, we engineered both single‐ and dual‐site mutations within the *ZmACC1*/*2* genes. Our herbicide‐resistance assays indicated that specific amino acid substitutions in the CT domain of ACCase, impairing herbicide binding affinity, confer varying levels of herbicide resistance. This aligns with prior studies that reported differential resistance profiles among ACCase mutants in rice (Liu et al., 2020; Zhang et al., 2023). Additionally, compared with the *ZmACC1* precise editing mutants, *ZmACC2* editing mutants exhibited significantly higher levels of herbicide resistance. Subsequent analysis of the expression data from various tissues demonstrated that *ZmACC2* expression was slightly higher than *ZmACC1*, especially in leaves. We obtained premature termination mutations for *ZmACC1* and *ZmACC2* from CBE by‐products. Phenotypic analysis demonstrated that the *ZmACC2* pretermination mutant exhibited a distinct chlorotic phenotype in the leaves. In contrast, the *ZmACC1* mutant did not show significant growth differences from the wild‐type (data not shown). These findings suggested that *ZmACC2* plays a more pivotal role in this metabolic pathway than *ZmACC1*. In addition, homozygous mutants of C2090R in either *ZmACC1* or *ZmACC2* are not lethal in maize, unlike rice. Meanwhile, the dual‐gene mutations of both *ZmACC1* and *ZmACC2* resulted in lethality, suggesting functional redundancies in *ZmACC*'s role in maize fatty acid biosynthesis that warrant further investigation. The *ZmACC1/2* double mutants exhibited no significant growth penalty under 4× herbicide treatment when intercropped with soybean in the field and maintained acceptable fitness even under 8× herbicide application. Future studies focused on identifying additional high‐resistance mutation sites or enhancing the expression levels of resistant alleles may further improve herbicide tolerance, thereby providing more robust and resilient options for intercropping and crop rotation systems involving maize and dicotyledonous crops.

## MATERIALS AND METHODS

### Plasmid construction

The cytidine deaminases (evoAPOBEC1, evoFERNY, evoCDA1, and RrA3F), adenosine deaminases (TadA8e and Tad8.20) were codon‐optimized for maize, and synthesized commercially (GenScript, Nanjing, China) (Table S6). The cytidine deaminase, adenosine deaminase, nCas9 (D10A), UGI, and maize Ubiquitin1 promoter sequences were amplified and assembled into a *Hin*dIII‐ and *Eco*911‐digested pCAMBIA3301 vector using NEBuilder® HiFi DNA Assembly Master Mix (NEB, Ipswich, MA, USA), producing P3301‐BE backbones, esgRNA transcription was driven by *ZmU6‐2* (Qi et al., 2018) and integrated with the EnSFR (*ZmESP*‐DsRED‐Nos) or EmSFR (*HvASP*‐eGFP‐Nos) expression cassette, which were amplified and assembled into *Sma*I‐ and *Hin*dIII‐digested P3301‐BE vectors. For triple target assembly, the complete target‐esgRNA scaffold sequence was synthesized commercially and driven by *35S‐CmYLCV‐ZmU6‐2* (Jiang et al., 2020). All PCRs were performed using a KAPA HiFi HotStart Ready MixPCR Kit (KAPA Biosystem, Cape Town, South Africa) with primers listed in Table S5. Construct details are listed in Figure S2.

### Transient transformation of maize protoplasts

For protoplast isolation, 14‐d‐old etiolated maize seedlings were sectioned into 1‐mm strips with a sterile blade. Tissue fragments were transferred into 20 mL of enzymatic digestion solution (pH 5.7) containing 0.8 M mannitol, 10 mM MES, 1.5% (w/v) cellulase R‐10, 0.4% (w/v) macerozyme R‐10, 10 mM CaCl_2_, and 0.1% (v/v) bovine serum albumin (BSA). After vacuum infiltration at −500 mbar for 30 min in the dark, the samples were incubated with gentle shaking (50 rpm) at 28°C for 4 h. The resulting protoplast suspension was filtered through a 70 μm nylon mesh and pelleted by centrifugation at 100 *g* for 5 min. The pellet was resuspended in W5 buffer (154 mM NaCl, 125 mM CaCl_2_, 5 mM KCl, 2 mM MES, pH 5.7) and rinsed three times via repeated centrifugation–resuspension cycles. After final resuspension in W5 buffer, the protoplasts were maintained on ice for 30 min. Cell density was adjusted to 5 × 10^5^ cells/mL using a hemocytometer.

For transformation, protoplasts were obtained and resuspended in MMG buffer (0.4 M mannitol, 15 mM MgCl_2_, 4 mM MES, pH 5.7). The transfection mixture was prepared in a 2 mL microcentrifuge tube by sequentially adding 200 μL protoplast suspension, 20 μL plasmid DNA (2 mg/mL), and 220 μL PEG solution (40% (w/v) PEG 4000, 0.2 M mannitol, 0.1 M CaCl_2_), with gentle mixing following each addition. After an 18‐min incubation at room temperature, the reaction was terminated by adding 880 μL W5 buffer. The transfected protoplasts were collected by centrifugation (100 *g*, 3 min), resuspended in 1 mL of fresh W5 buffer, and transferred to 24‐well culture plates. Cultures were maintained at 28°C in the dark for 72 h before analysis.

### DNA isolation and mutation detection

Plant genomic DNA was isolated from transformant leaves (or protoplasts) using the cetyltrimethylammonium bromide (CTAB) method. PCR amplification of genomic targets and BE transgenes was conducted using specific primers outlined in Table S5. The PCR products were deep sequenced using the Hi‐TOM platform (Liu et al., 2019) (http://www.hi-tom.net/hi-tom/) or Sanger sequencing to detect potential mutations.

### *Agrobacterium*‐mediated transformation

All constructs for maize transformation were introduced into *Agrobacterium tumefaciens* strain EHA105. The *Agrobacterium*‐mediated maize transformation was performed using immature inbred KN5585 embryos following a commercial service provided by Weimi Biotechnology Co. Ltd (Changzhou, Jiangsu, China). Before transformation, equal volumes of *Agrobacterium* suspensions carrying CBE‐A and CBE‐C constructs were mixed at a 1:1 ratio (v/v). Similarly, *Agrobacterium* cultures containing CBE‐F and CBE‐R constructs were proportionally combined in a 1:1 volumetric ratio to prepare the final inoculation mixture.

### Imaging and fluorescence assessment

Maize seed kernels, ears, seedlings, and whole plants were photographed using a Canon 70D digital camera (Canon, Tokyo, Japan). The ears and kernels of various BEs transformants were characterized based on En‐SFR/Em‐SFR markers with a LUYOR‐3425RG fluorescence flashlight (LUYOR, Upland, CA, USA) with a 550 nm excitation wavelength with a DsRED‐specific filter and a 480 nm excitation wavelength with an eGFP‐specific filter (Dong et al., 2018). T1 seeds lacking SFR markers were selected for further genotyping and phenotyping.

### Herbicide‐resistance tests for ACCase mutants

Seeds from wild‐type and ZmACC1^P1831A^, ZmACC1^P1831L^, ZmACC1^P1831S^, ZmACC2^P1831F^, ZmACC1^P1831L^ZmACC2^P1831F^, ZmACC1^C2090R/Y2091H^, ZmACC2^C2090R^, ZmACC2^C2090R/Y2091H^, and ZmACC1^C2090R/Y2091H‐He^ZmACC2^C2090R^ mutants were grown to the two‐to‐three‐leaf stage in a greenhouse under 16‐h day length at 28°C and 8‐h darkness at 25°C, and then sprayed with varying concentrations of ZmACCase inhibitors. The concentrations included 1× dosage: 13.0 g a.i./ha fluazifop‐p‐butyl, 12.6 g a.i./ha quizalofop‐p‐ethyl, 12.2 g a.i./ha clethodim and 13.5 g a.i./ha pinoxaden. Phenotypes were observed at 14 DAS.

To test herbicide resistance in the field, ZmACC1^P1831L^ZmACC2^P1831F^ homozygous mutants and wild‐type controls were intercropped with soybean variety Zhonghuang301, then treated with two, four, and eightfold quizalofop‐p‐ethyl and clethodim at the four‐ to five‐leaf stage. Phenotypic data were obtained at 12 and 26 day post herbicide treatment.

### Statistical analyses

SPSS software was used for statistical evaluation of phenotypic data. An independent samples *t*‐test was used to compare the means of mutant and wild‐type samples. This test can assess differences between two groups when the samples are independent. The threshold for statistical significance was established at *P* < 0.05.

## CONFLICT OF INTEREST

The authors declare that they have no conflicts of interest.

## AUTHOR CONTRIBUTIONS

X.F. and N.W. performed the majority of the research. X.F. and L.L. drafted the manuscript. X.F., L.L., and D.Q. revised the manuscript, while X.Q., Z.G., and C.L. provided critical feedback. J.Z. and C.X. designed the experiments, supervised the study, and edited the manuscript. All authors read and approved the final version.

## Supporting information

Additional Supporting Information may be found online in the supporting information tab for this article: http://onlinelibrary.wiley.com/doi/10.1111/jipb.13964/suppinfo

**Figure S1.** Editing efficiency and windows for different base editors (Bes) at *ZmACC1/2* targets in maize protoplast cells
**Figure S2.** Vector details for cytosine base editor (CBE) and adenine base editors (ABE) elements used in stable transformation
**Figure S3.** Gel electrophoresis diagram of transgenic elements detection for evoAPOBEC1, evoCDA1, evoFERNY, and RrA3F
**Figure S4.** Gel electrophoresis diagram of transgenic elements detection for TadA8e and TadA8.20
**Figure S5.** Field phenotyping of ZmACC1^P1831L^ZmACC2^P1831F^ homozygous mutants in response to quizalofop‐p‐ethyl and clethodim treatments
**Figure S6.** Field phenotyping of ZmACC1^P1831L^ZmACC2^P1831F^ homozygous mutants in response to clethodim treatments
**Table S1.** Target site sequences were edited with different base editors (BEs) in maize protoplast testing
**Table S2.** The genotypes of four endogenous targets produced by CBE‐evoCDA1 in the T0 generation
**Table S3.** Genetic characterization and inheritance of T0 *ZmACC1*/*2* mutants (P1831, C2090, and Y2091) over generations
**Table S4.** Analysis of potential off‐target impacts of different base editors (BEs)
**Table S5.** List of primers utilized in this study
**Table S6.** The sequences of the gene fragments for evoAPOBEC1, evoCDA1, evoFERNY, RrA3F, TadA8.20, TadA8e and the tGly‐T1‐esgRNA scaffold‐HDV‐tMet structure
