# Supplementary material for: Development of cytosine and adenine base editors for maize precision breeding
Source: J Integr Plant Biol. 2025 Jul 11;67(10):2731–43. doi: 10.1111/jipb.13964 (PMC12498073; doi:10.1111/jipb.13964)
Supplement: Supplementary file 1 — Figure S1. Editing efficiency and windows for different base editors (Bes) at ZmACC1/2 targets in maize protoplast cells Figure S2. Vector details for cytosine base editor (CBE) and adenine base editors (ABE) elements used in stable transformation Figure S3. Gel electrophoresis diagram of transgenic elements detection for evoAPOBEC1, evoCDA1, evoFERNY, and RrA3F Figure S4. Gel electrophoresis diagram of transgenic elements detection for TadA8e and TadA8.20 Figure S5. Field phenotyping of ZmACC1P1831LZmACC2P1831F homozygous mutants in response to quizalofop‐p‐ethyl and clethodim treatments Figure S6. Field phenotyping of ZmACC1P1831LZmACC2P1831F homozygous mutants in response to clethodim treatments Table S1. Target site sequences were edited with different base editors (BEs) in maize protoplast testing Table S2. The genotypes of four endogenous targets produced by CBE‐evoCDA1 in the T0 generation Table S3. Genetic characterization and inheritance of T0 ZmACC1/2 mutants (P1831, C2090, and Y2091) over generations Table S4. Analysis of potential off‐target impacts of different base editors (BEs) Table S5. List of primers utilized in this study Table S6. The sequences of the gene fragments for evoAPOBEC1, evoCDA1, evoFERNY, RrA3F, TadA8.20, TadA8e and the tGly‐T1‐esgRNA scaffold‐HDV‐tMet structure [file JIPB-67-2731-s001.docx]

**TEMPLATE FOR JIPB SUPPORTING INFORMATION**

**Development of Cytosine and Adenine Base Editors for Maize Precision Breeding**

Xiao Fu ^1^, Nan Wang ^1^, Lina Li ^1, 2^, Dexin Qiao ^1^, Xiantao Qi ^1^, Changlin Liu ^1, 2^, Zhaoxu Gao ^1^, Chuanxiao Xie ^1, 2, *^, Jinjie Zhu ^1, *^

^1^ State Key Laboratory of Crop Gene Resources and Breeding, Institute of Crop Sciences, Chinese Academy of Agricultural Sciences, Beijing 100081, China.

^2^ National Nanfan Research Institute (Sanya), Chinese Academy of Agricultural Sciences, Sanya, Hainan 572025, China.

**SUPPORTING INFORMATION**

**Figure S1.** Editing efficiency and windows for different BEs at *ZmACC1/2* targets in maize protoplast cells.

**Figure S2.** Vector details for CBE and ABE elements used in stable transformation.

**Figure S3.** Gel electrophoresis diagram of transgenic elements detection for evoAPOBEC1, evoCDA1, evoFERNY, and RrA3F.

**Figure S4.** Gel electrophoresis diagram of transgenic elements detection for TadA8e and TadA8.20.

**Figure S5.** Field phenotyping of ZmACC1^P1831L^ZmACC2^P1831F^ homozygous mutants in response to quizalofop-p-ethyl and clethodim treatments.

**Figure S6.** Field phenotyping of ZmACC1^P1831L^ZmACC2^P1831F^ homozygous mutants in response to clethodim treatments.

**Table S1.** Target site sequences were edited with different BEs in maize protoplast testing.

**Table S2.** The genotypes of four endogenous targets produced by CBE-evoCDA1 in the T0 generation.

**Table S3.** Genetic characterization and inheritance of T0 *ZmACC1*/*2* mutants (P1831, C2090, and Y2091) over generations.

**Table S4.** Analysis of potential off-target impacts of different BEs.

**Table S5.** List of primers utilized in this study.

**Table S6.** The sequences of the gene fragments for evoAPOBEC1, evoCDA1, evoFERNY, RrA3F, TadA8.20, TadA8e and the tGly-T1-esgRNA scaffold-HDV-tMet structure.


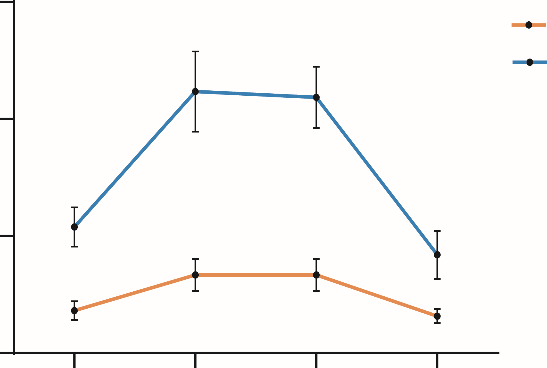


C to T editing efficiency (%)

0

16

8

24

C to T editing efficiency (%)

0

16

8

24

evoAPOBEC1

evoCDA1

evoFERNY

RrA3F

A

C

F

R

C3

C4

C7

C8

C to T editing efficiency (%)

0

16

8

24

C to T editing efficiency (%)

0

16

8

24

evoAPOBEC1

evoCDA1

evoFERNY

RrA3F

A

C

F

R

C3

C4

C7

C8


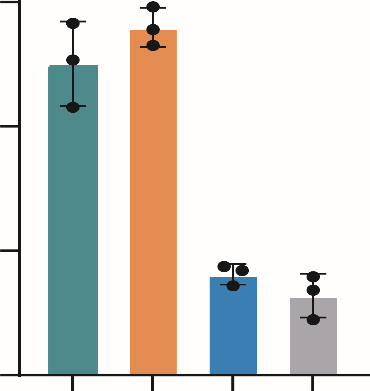

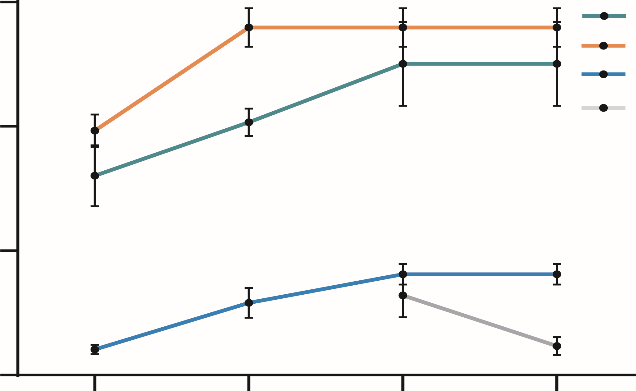

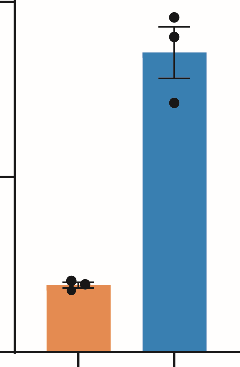


0

2.5

5.0


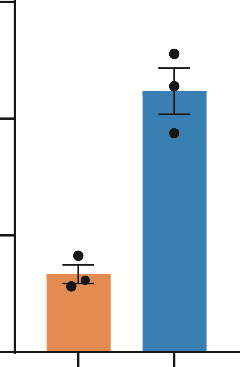


0

2

4

6

TadA8.20

TadA8e

A to G editing efficiency (%)

A to G editing efficiency (%)


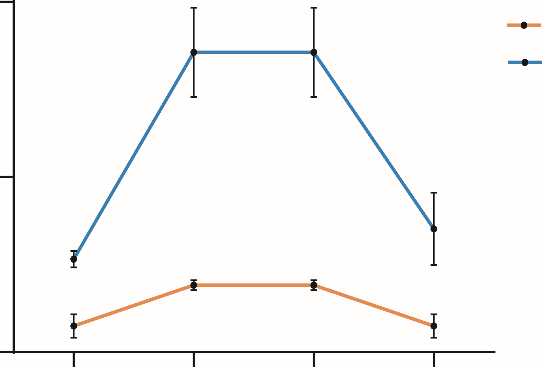


0

2.5

5.0

A to G editing efficiency (%)

TadA8.20

TadA8e

TadA8.20

TadA8e

A3

A5

A6

A8

TadA8.20

TadA8e

A3

A5

A6

A8

0

2

4

6

A to G editing efficiency (%)

**A**

**B**

**C**

**D**

**E**

**F**

**G**

**H**


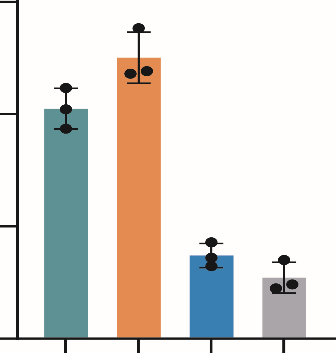

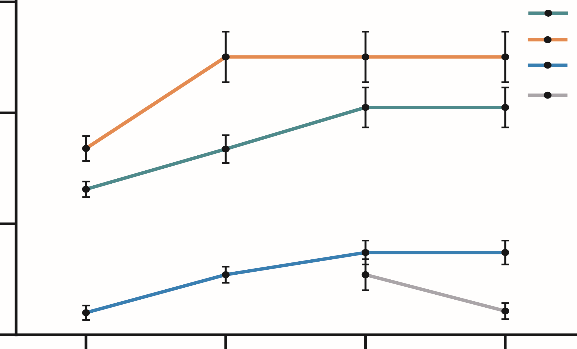


Figure S1. Editing efficiency and windows for different BEs at *ZmACC1/2* targets in maize protoplast cells.

(A-B) Editing Efficiency and Activity Window Analysis of CBE-evoAPOBEC1, evoCDA1, evoFERNY and RrA3F at Target Site *ZmACC1*-P1831 in Maize Protoplasts.

(C-D) Editing Efficiency and Activity Window Analysis of CBE-evoAPOBEC1, evoCDA1, evoFERNY and RrA3F at Target Site *ZmACC2-*P1831 in Maize Protoplasts.

(E-F) Editing Efficiency and Activity Window Analysis of ABE-TadA8.20 and TadA8e at Target Site *ZmACC1*-C2090R, Y2091 in Maize Protoplasts.

(G-H) Editing Efficiency and Activity Window Analysis of ABE-TadA8.20 and TadA8e at Target Site *ZmACC2*-C2090R, Y2091 in Maize Protoplasts.


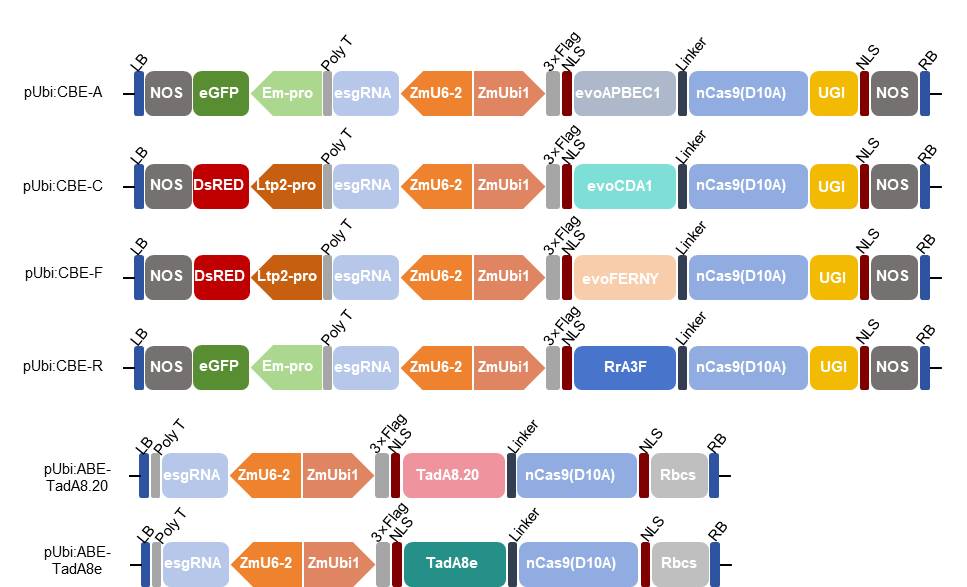


Figure S2. Vector details for CBE and ABE elements used in stable transformation.

Architectures of pUbi: CBE-A, pUbi: CBE-C, pUbi: CBE-F, pUbi: CBE-R, pUbi: ABE-TadA8.20 and pUbi: ABE-TadA8e. Linker, a 32-aa linker; 3×Flag, a consists of three repeated Flag peptide sequences. NLS, Nuclear Localization Signal.


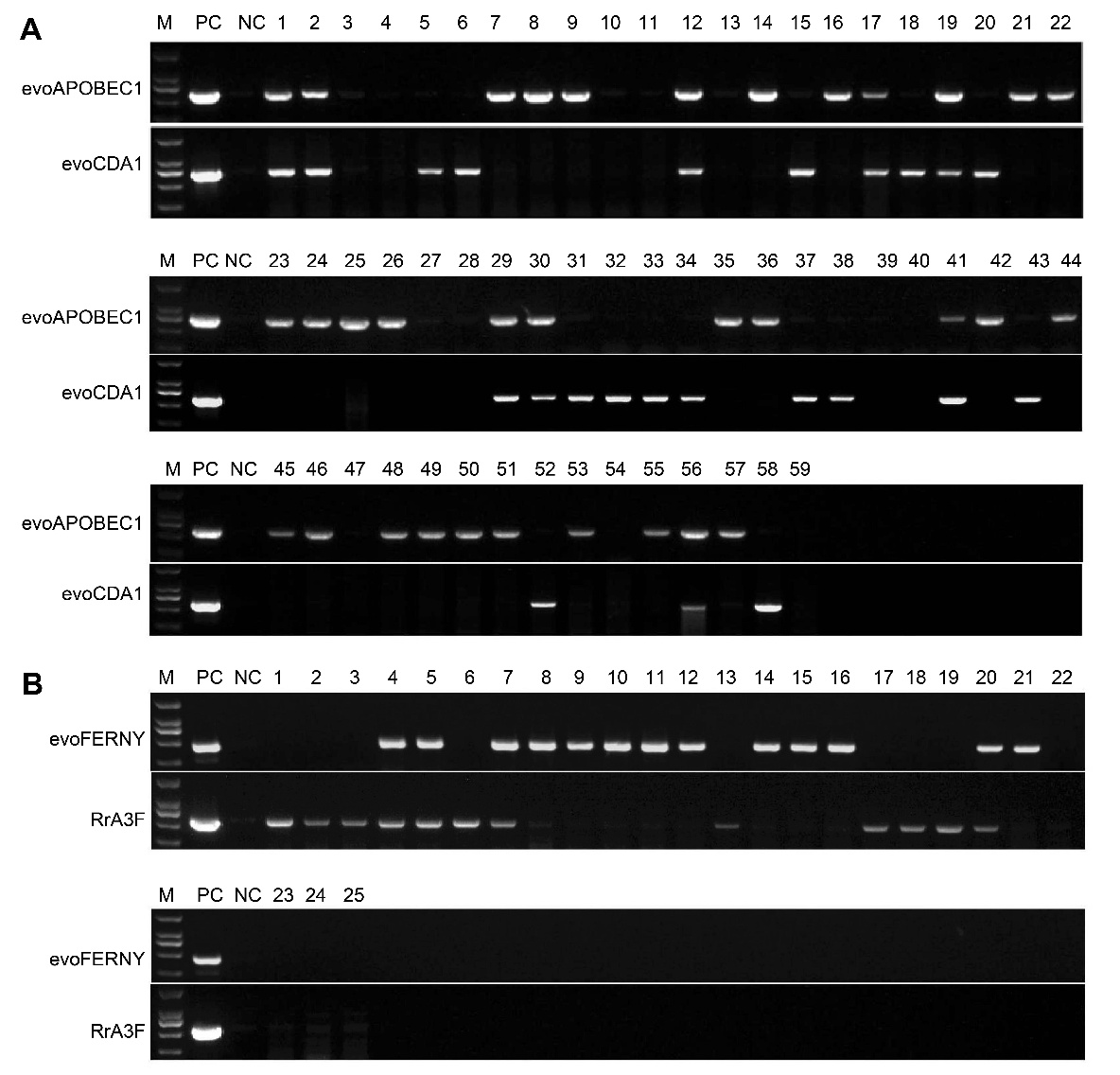


Figure S3. Gel electrophoresis diagram of transgenic elements detection for evoAPOBEC1, evoCDA1 , evoFERNY and RrA3F.

(A) Transgenic element detection of evoAPOBEC1 and evoCDA1 in T0 maize co-transformation lines.

(B) Transgenic element detection of evoFERNY and RrA3F in T0 maize co-transformation lines. M, marker; PC, positive control; NC, negative control.


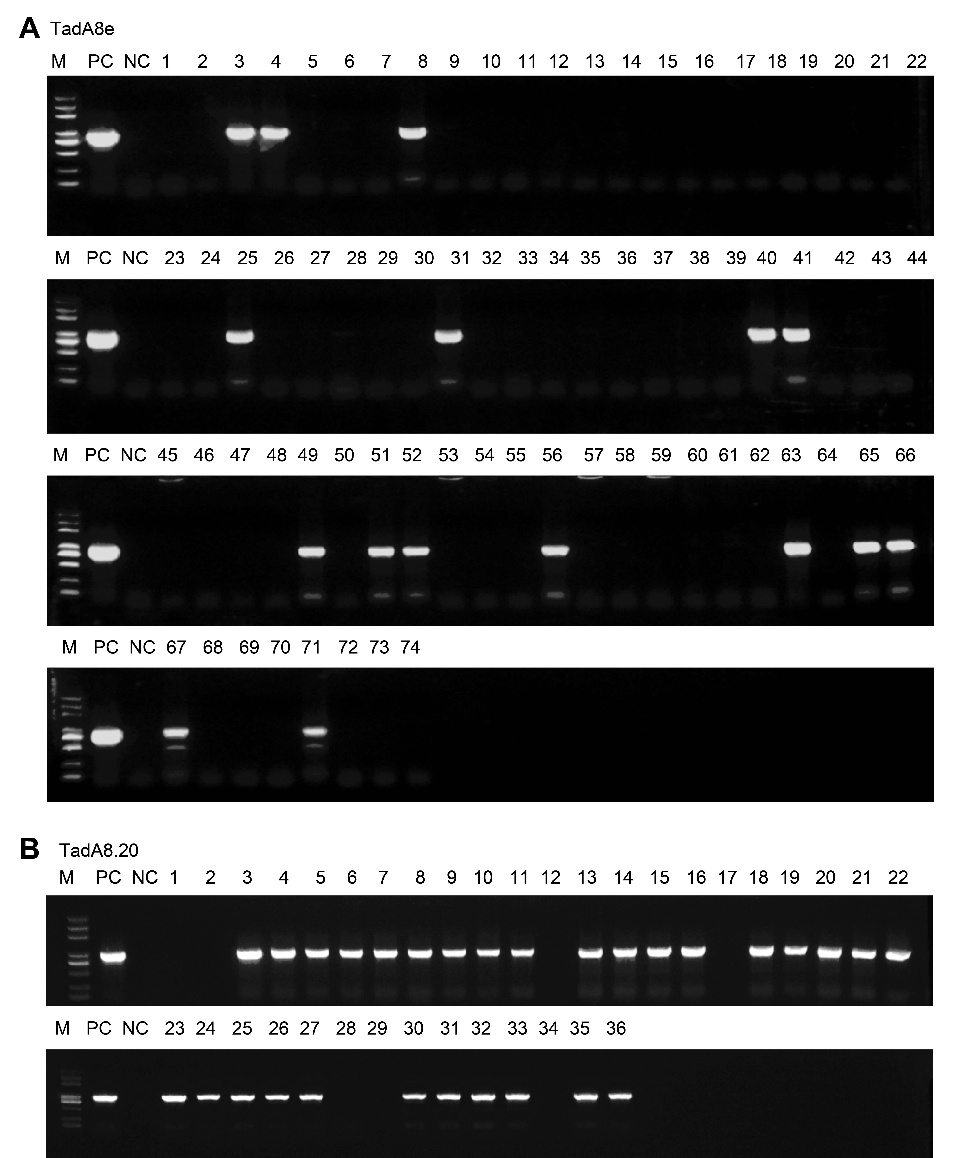


Figure S4. Gel electrophoresis diagram of transgenic elements detection for TadA8e and TadA8.20.

(A) Transgenic element detection of TadA8e in T0 maize co-transformation lines.

(B) Transgenic element detection of TadA8.20 in T0 maize co-transformation lines. M, marker; PC, positive control; NC, negative control.


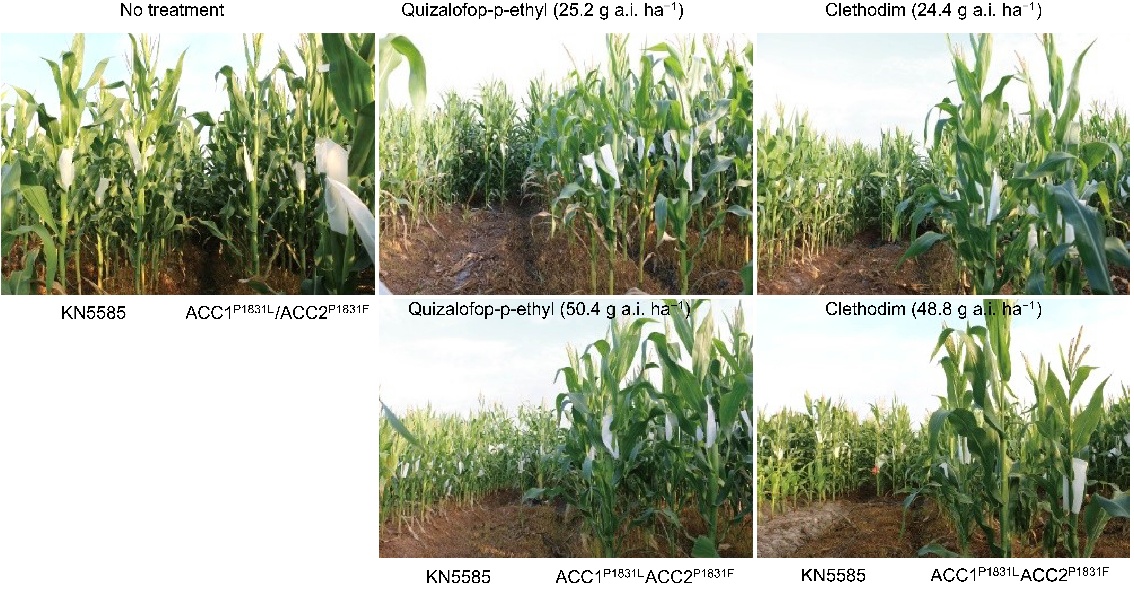


Figure S5. Field phenotyping of ACC1^P1831L^ACC2^P1831F^ homozygous mutants in response to quizalofop-p-ethyl and clethodim treatments.

The herbicide resistance performance of both wild-type and ACC1^P1831L^ACC2^P1831F^ plants following treatments with quizalofop-p-ethyl at concentrations of 25.2 and 50.4 g a.i. ha^−1^, and clethodim at concentrations of 24.4 and 48.8 g a.i. ha^−1^, administered at the six-leaf stage. Phenotypic data were collected 26 days after herbicide application.


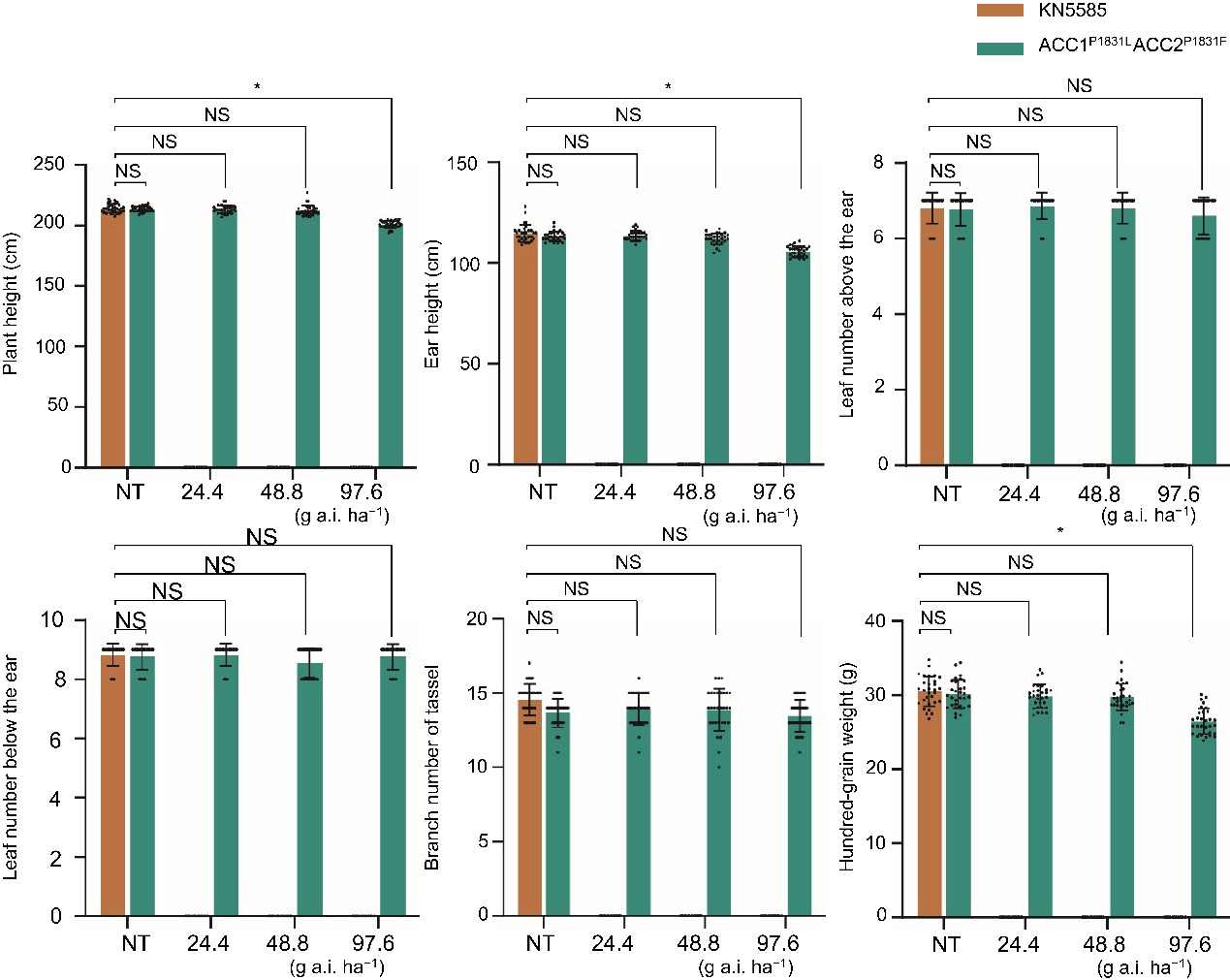


Figure S6. Field phenotyping of ACC1^P1831L^ACC2^P1831F^ homozygous mutants in response to clethodim treatments.

Comparative analysis of agronomic traits between the wild type and the ACC1^P1831L^ACC2^P1831F^ mutant, both with and without clethodim treatment. The data of plant height, ear height, number of leaves above the ear, number of leaves below the ear, number of tassel branches and hundred-grain weight were collected and conducted for significance analysis (P < 0.05). Displayed values represent means ± standard deviation (n = 30).

Table S1. Target site sequences edited with different BEs in maize protoplast testing.

| Target sites | Target sequence (5’-3’) |
| --- | --- |
| *ACC1/2*-P1831 | GACCAGCCTATTATTTTAACAGG |
| *ACC1/2*-C2090, Y2901 | GCATAACACTCAATGCGGTCTGG |
| TC1 | TCTCATCATCTTCATTATCGAGG |
| TC2 | CGTACTCGTTCACTCCGTGATGG |
| TC3 | TGGGCTCAACGACATTGGTATGG |
| CC1 | ATTAGCCGCAGCTGCCTCAAAGG |
| CC2 | AATCGCCAGAGAAGTTCAGCTGG |
| CC3 | GCTCCCCAATATTTGTCATGAGG |
| GC1 | TTTCTGCTGTTTGGCCTGGTTGG |
| GC2 | AAACTGCACTGCAGAAGTCATGG |
| GC3 | CATAAGCTGGAGCTAGATAGTGG |
| AC1 | TGGCAACTCCGGAAGACATGAGG |
| AC2 | CAAGTACAAGGTGAAGTCCCTGG |
| AC3 | TATTGACTCTGTTGTGGGCAAGG |
| ABE-T1 | ACGAAGGCAGTCCTGAACGAGGG |
| ABE-T2 | CCTGAACAAGCTCCTTGGGCGGG |
| ABE-T3 | AAATAGTAAATCCTACGTGAAGG |

Table S2. The genotypes of four endogenous targets produced by CBE-evoCDA1 in the T0 generation.

| T0 lines | Site-T1 | | | Site-T2 | | | Site-T3-1 | | | Site-T3-2 | | |
| --- | --- | --- | --- | --- | --- | --- | --- | --- | --- | --- | --- | --- |
|  | Base mutations | Amino acid mutations | Genotype | Base mutations | Amino acid mutations | Genotype | Base mutations | Amino acid mutations | Genotype | Base mutations | Amino acid mutations | Genotype |
| 1 | WT | WT |  | WT | WT |  | WT | WT |  | WT | WT | WT |
| 2 | AGA->AAA，GGA->AAA | R333K, G334K | Ho | AGA->AAA，GGT->AAT | R322K, G323N | Ho | AGA->AAA, GAA->AAA, GGC->AAC | R363K, E361K, G364N | Bi | AGA->AAA, GGC->AAC | R363K, G364N | Ho |
| 3 | AGA->AAA，GGA->AAA | R333K, G334K | Ho | AGA->AAA，GGT->AAT | R322K, G324N | Ho | AGA->AAA, GAA->AAA | R363K, G364N | Ho | AGA->AAA, GGC->AAC | R363K, G364N | Ho |
| 4 | AGA->AAA，GGA->AAA | R333K, G334K | Ho | AGA->AAA，GGT->AAT | R322K, G325N | Ho | AGA->AAA, GAA->AAA | R363K, G364N | Ho | AGA->AAA, GGC->AAC | R363K, G364N | Ho |
| 5 | AGA->AAA，GGA->AAA | R333K, G334K | Ho | AGA->AAA，GGT->AAT | R322K, G326N | Ho | AGA->AAA, GAA->AAA, GGC->AAC | R363K, E361K, G364N | Bi | AGA->AAA, GGC->AAC | R363K, G364N | Ho |
| 6 | AGA->AAA，GGA->AAA | R333K, G334K | Ho | AGA->AAA，GGT->AAT | R322K, G327N | Ho | AGA->AAA, GAA->AAA, GGC->AAC | R363K, E361K, G364N | Bi | AGA->AAA, GGC->AAC | R363K, G364N | Ho |
| 7 | AGA->AAA，GGA->AAA | R333K, G334K | Ho | AGA->AAA，GGT->AAT | R322K, G328N | Ho | AGA->AAA, GAA->AAA | R363K, G364N | Ho | AGA->AAA, GGC->AAC | R363K, G364N | Bi |
| 8 | AGA->AAA，GGA->AAA | R333K, G334K | Ho | AGA->AAA，GGT->AAT | R322K, G329N | Ho | AGA->AAA, GAA->AAA | R363K, G364N | Ho | AGA->AAA, GGC->AAC | R363K, G364N | Bi |
| 9 | AGA->AAA，GGA->AAA | R333K, G334K | Ho | AGA->AAA，GGT->AAT | R322K, G330N | Ho | AGA->AAA, GAA->AAA, GGC->AAC | R363K, E361K, G364N | Bi | WT | WT | WT |
| 10 | AGA->AAA, GGA->AAA | R333K, G334K | Ho | AGA->AAA | R322K | Ho | AGA->AAA, GAA->AAA | R363K, G364N | Ho | AGA->AAA, GGC->AAC | R363K, G364N | Bi |
| 11 | AGA->ATA，AGA->AAA | R333I, R333K, G334K | Bi | AGA->AAA | R322K | He | -- | -- | Chi | AGA->AAA, GGC->AAC | R363K, G364N | He |
| 12 | AGA->AAA | R333K | Ho | AGA->AAA | R322K | Ho | AGA->AAA, GAA->AAA | R363K, G364N | Ho | AGA->AAA, GGC->AAC | R363K, G364N | Ho |
| 13 | AGA->AAA | R333K | Bi | AGA->AAA | R322K | Ho | AGA->AAA, GAA->AAA | R363K, G364N | Ho | AGA->AAA, GGC->AAC | R363K, G364N | Ho |
| 14 | AGA->AAA | R333K | Ho | AGA->AAA | R322K | Ho | AGA->AAA, GAA->AAA, GGC->AAC | R363K, E361K, G364N | Bi | AGA->AAA, GGC->AAC | R363K, G364N | Ho |
| 15 | AGA->AAA | R333K | Ho | AGA->AAA | R322K | Ho | AGA->AAA, GAA->AAA | R363K, G364N | Ho | AGA->AAA, GGC->AAC | R363K, G364N | Bi |
| 16 | AGA->AAA | R333K | Ho | AGA->AAA | R322K | Ho | AGA->AAA, GAA->AAA | R363K, G364N | Ho | AGA->AAA, GGC->AAC | R363K, G364N | Bi |
| 17 | WT | WT | WT | WT | WT |  | WT | WT | WT | WT | WT | WT |
| 18 | AGA->AAA | R333K | Ho | AGA->AAA | R322K | Ho | AGA->AAA, GAA->AAA, GGC->AAC | R363K, E361K, G364N | Bi | AGA->AAA, GGC->AAC | R363K, G364N | Bi |
| 19 | WT | WT | WT | WT | WT |  | AGA->AAA, GGC->AAC | R363K, G364N | He | WT | WT | WT |

Note: Ho-Homozygous, He-Heterozygous, Bi-Biallelic, Chi-Chimeric.

Table S3. Genetic characterization and inheritance of T0 *ZmACC1/2* mutants (P1831, C2090, and Y2091) over generations.

| Target | Line No. | T0 generation | | | | T1 generation | | | | T2 generation |
| --- | --- | --- | --- | --- | --- | --- | --- | --- | --- | --- |
|  |  | *ZmACC1* | | *ZmACC2* | | *ZmACC1* | | *ZmACC2* | | Mutant |
|  |  | Base mutations | Amino acid mutations,  Genotype | Base mutations | Amino acid mutations,  Genotype | Base mutations | Amino acid mutations,  Genotype | Base mutations | Amino acid mutations,  Genotype |  |
| P1831  (CCT) | 01^#^ | GCT; TCT | P1831A;  P1831S, Bi | GCT; TCT | P1831A;  P1831S, Bi | GCT; TCT | P1831A;  P1831S, HE | GCT; TCT | P1831A;  P1831S, HE | ZmACC1^P1831A^HO |
|  | 06^#^ | TCT | P1831S, HE | TCT | P1831S, HE | TCT | P1831S, HE | TCT | P1831S, HE | ZmACC1^P1831S^HO |
|  | 14^#^ | CTT | P1831L, HE | TTT | P1831F, HE | CTT | P1831L, HE | TTT | P1831F, HE | ZmACC1^P1831L^HO |
|  |  |  |  |  |  |  |  |  |  | ZmACC1^P1831L^  ZmACC2^P1831F^HO |
|  | 37^#^ | WT | WT | TTT | P1831F, HE | WT | WT | TTT | P1831F, HE | ZmACC2^P1831F^HO |
| C2090  (ACA) | 42^#^ | WT | WT | GCA | C2090R, HE | WT | WT | GCA | C2090R, HE | ZmACC2^C2090R^HO |
| C2090  (ACA)  Y2091  (ATA) | 33^#^ | ACA/GTA | Y2091H, HE | GCG/GTA | C2090R,  Y2091H, HE | ACA/GTA | Y2091H, HE | GCG/GTA | C2090R,  Y2091H, HE | ZmACC2^C2090R/Y2091H^HO |
|  | 46^#^ | GCG/GTA | C2090R,  Y2091H, HE | ACA/GTA | Y2091H, HE | GCG/GTA | C2090R,  Y2091H, HE | ACA/GTA | Y2091H, HE | ZmACC1^C2090R/Y2091H^HO |

Note: mutated bases are marked in red. Ho-Homozygous, He-Heterozygous, Bi-Biallelic.

Table S4. Analysis of potential off-target impacts of different BEs.

The PAM sequences highlighted in black and the mismatches to the target sequence in the potential off-target region are in red, respectively.

| Target | Putative off-target locus | Sequence of the putative  off-target site | No. of mismatching bases | No. of plants sequenced | No. of plant with mutation |
| --- | --- | --- | --- | --- | --- |
| *ZmACC1/2*-P1831 | Zm00001d014294(5: -39805678) | GA**T**CA**A**CC**A**ATTATTTTGACTGG | 3 | 55 (A:24; C:14; F:9; R:8) | 0 |
|  | Zm00001d012641(8: +177676363) | G**G**CCAGCC**AT**TT**T**TTTTAACCGG | 4 | 55 (A:24; C:14; F:9; R:8) | 0 |
| *ZmACC1/2*-C2090, Y2091 | Zm00001d040624(3: +54718814) | GCAT**GC**C**T**CTCAAT**C**CGGTCGGG | 4 | 45 (TadA8e:16; TadA8.20:29) | 0 |
|  | Zm00001d015699(5: -108854400) | GCA**A**AA**A**ACTC**T**ATGC**T**GTCAGG | 4 | 45 (TadA8e:16; TadA8.20:29) | 0 |
|  | Zm00001d039630(3: -9560915) | GCA**A**AA**A**ACTC**T**ATGC**T**GTCAGG | 4 | 45 (TadA8e:16; TadA8.20:29) | 0 |
| Site-T1 | Zm00001d047175(9: -120986138) | G**C**CCTC**AC**GT**A**TCAGGAAGAGGG | 4 | 19 | 0 |
|  | Zm00001d046039(9: +58133575) | **AG**CCT**A**TTGTTT**T**AGGAAGAAGG | 4 | 19 | 0 |
|  | Zm00001d048147(9: +150861468) | GT**TT**TCTTGTTTC**T**GGAAGATGG | 3 | 19 | 0 |
| Site-T2 | Zm00001d047912(9: -145087204) | GA**T**CTCTG**C**TTTCCGGT**CT**GTGG | 4 | 19 | 0 |
| Site-T3-1/T3-2 | Zm00001d026249(10: +141899917) | **A**GCCT**T**TTGTTTCA**A**GAA**A**GCGG | 4 | 19 | 0 |
|  | Zm00001d046039(9: +58133575) | **A**GCCT**A**TTGTTT**T**AGGAAG**A**AGG | 4 | 19 | 0 |
|  | Zm00001d023384(10: -4335658) | GGCCT**TG**TG**C**T**C**CAGGAAGGTGG | 4 | 19 | 0 |
|  | Zm00001d049210(4: +20791692) | GGC**AA**CTTGT**A**T**T**AGGAAGGTGG | 4 | 19 | 0 |
|  | Zm00001d043434(3: +199747734) | GGC**AA**CTTGT**A**T**T**AGGAAGGTGG | 4 | 19 | 0 |
|  | Zm00001d047946(9: +145943077) | GGC**AA**CTTGT**A**T**T**AGGAAGGTGG | 4 | 19 | 0 |
|  | Zm00001d043327(3: +196603070) | GGC**AA**CTTGT**A**T**T**AGGAAGGTGG | 4 | 19 | 0 |
|  | Zm00001d024998(10: +98549404) | GGC**AA**CTTGT**A**T**T**AGGAAGGTGG | 4 | 19 | 0 |
|  | Zm00001d049821(4: +46290069 | GGC**AA**CTTGT**A**T**T**AGGAAGGTGG | 4 | 19 | 0 |
|  | Zm00001d049314(4: +25986019) | GGC**AA**CTTGT**A**T**T**AGGAAGGTGG | 4 | 19 | 0 |
|  | Zm00001d049313(4: +25986019) | GGC**AA**CTTGT**A**T**T**AGGAAGGTGG | 4 | 19 | 0 |

Table S5. List of primers utilized in this study.

| Primer name | Primer sequence (5’ - 3’) | Used for |
| --- | --- | --- |
| HF-TC1 | GGAGTGAGTACGGTGTGCCCTAATCGCCAGAGAAGTTC | Site TC1 |
| HR-TC1 | GAGTTGGATGCTGGATGGGACGAACAGACTGGCTGTGC |  |
| HF-TC2 | GGAGTGAGTACGGTGTGCCCTAATCGCCAGAGAAGTTC | Site TC2 |
| HR-TC2 | GAGTTGGATGCTGGATGGTAGCTTGTATCAAAAGATA |  |
| HF-TC3 | GGAGTGAGTACGGTGTGCCAGCGTCTTGACCAGCCTAT | Site TC3 |
| HR-TC3 | GAGTTGGATGCTGGATGGTCCACCAATGTTTGCAGGAA |  |
| HF-CC1/2 | GGAGTGAGTACGGTGTGCCTTCAATTGTGCTGTCTGGG | Site CC1/2 |
| HR-CC1/2 | GAGTTGGATGCTGGATGGCCTCGATAATGAAGATGATG |  |
| HF-CC3 | GGAGTGAGTACGGTGTGCCTGGCAGGTTCATAATGATG | Site CC3 |
| HR-CC3 | GAGTTGGATGCTGGATGGACTAAAGGTGAAAAGGTATGG |  |
| HF-GC1 | GGAGTGAGTACGGTGTGCTTGTGGAAGTCCAATTGTCA | Site GC1 |
| HR-GC1 | GAGTTGGATGCTGGATGGTTGCGGTCAATACATCTGGC |  |
| HF-GC2 | GGAGTGAGTACGGTGTGCCCTAAGCTCATAATGATGG | Site GC2 |
| HR-GC2 | GAGTTGGATGCTGGATGGATCGGAATTATAGGAGTGCC |  |
| HF-GC3 | GGAGTGAGTACGGTGTGCGACTGAAGAAGACTATGCTCGC | Site GC3 |
| HR-GC3 | GAGTTGGATGCTGGATGGAGCACTGGCAATAGCAGCAC |  |
| HF-AC1 | GGAGTGAGTACGGTGTGCGGACATGGGCTAATGATAC | Site AC1 |
| HR-AC1 | GAGTTGGATGCTGGATGGGAGTTGAACATTGGCGTAGT |  |
| HF-AC2 | GGAGTGAGTACGGTGTGCACAATGGAATGGCAGCAGCA | Site AC2 |
| HR-AC2 | GAGTTGGATGCTGGATGGGGCACCTCTACGAATTGGTC |  |
| HF-AC3 | GGAGTGAGTACGGTGTGCTGCTTATTCTAGGGCATATG | Site AC3 |
| HR-AC3 | GAGTTGGATGCTGGATGGCGCCCAAGGAGCTTGTTCAG |  |
| HF-AT1/2 | GGAGTGAGTACGGTGTGCCAGCGTCTTGACCAGCCTAT | Site ABE-AT1/2 |
| HR-AT1/2 | GAGTTGGATGCTGGATGGTCCACCAATGTTTGCAGGAA |  |
| HF-AT3 | GGAGTGAGTACGGTGTGCCCTAAGCTCATAATGATGG | Site ABE-AT3 |
| HR-AT3 | GAGTTGGATGCTGGATGGATCGGAATTATAGGAGTGCC |  |
| OFT-JCF1 | TAGCTCACGAGCTGCAACTA | *Zm00001d014294* off-target site |
| OFT-JCR1 | CTACAGCTATGACACCAACT |  |
| OFT-JCF2 | GGCCGTCATGCGTCTTGATG | *Zm00001d012641* off-target site |
| OFT-JCR2 | CGGCGGTTCTTAGCAGTATG |  |
| OFT-JCF3 | GTCAGTGTCGGCGTATGTGT | *Zm00001d040624* off-target site |
| OFT-JCR3 | CTAGCGACAGATACATGCTA |  |
| OFT-JCF4 | GGGTGGACAGATGGTCCAAT | *Zm00001d015699* off-target site |
| OFT-JCR4 | TCATCTGGCACCATCTCTTC |  |
| OFT-JCF5 | GTAAGACAATAGACAAGCTAAGG | *Zm00001d039630* off-target site |
| OFT-JCR5 | CATCTGGCACCATCTCTTCA |  |
| OFT-JCF6 | CCTTGTGACTTCGTTCCTTA | *Zm00001d047175* off-target site |
| OFT-JCR6 | CTCGAATACGCAGGAGAACT |  |
| OFT-JCF7 | CCTATAGGTGCATAGGACAG | *Zm00001d046039* off-target site |
| OFT-JCR7 | CTTGATTCAGATGCAGATTG |  |
| OFT-JCF8 | GTAGGTCACGCGTGCTAGAA | *Zm00001d048147* off-target site |
| OFT-JCR8 | TCACTGAGCTCATCAAGTTC |  |
| OFT-JCF9 | GAGTTAGCGATGGCCGGAGA | *Zm00001d047912* off-target site |
| OFT-JCR9 | ATCACAACATTGGCCTGGAG |  |
| OFT-JCF10 | CTCCAGCCTTACATTGATTG | *Zm00001d026249* off-target site |
| OFT-JCR10 | TCGACATTGTGTTCAGACCG |  |
| OFT-JCF11 | CCTATAGGTGCATAGGACAG | *Zm00001d046039* off-target site |
| OFT-JCR11 | CTTGATTCAGATGCAGATTG |  |
| OFT-JCF12 | CCAAGCGCTGGATCACCAAG | *Zm00001d023384* off-target site |
| OFT-JCR12 | TCACGGCTTGCCCTTGTGCT |  |
| OFT-JCF13 | CGTAAACTTGTGGTGGCTGA | *Zm00001d049210* off-target site |
| OFT-JCR13 | TGACTACGCTCCTGCATGAA |  |
| OFT-JCF14 | CCATGCTTCTATCATGGAAC | *Zm00001d043434* off-target site |
| OFT-JCR14 | GTCCCAACATAATCCTGTGG |  |
| OFT-JCF15 | TCACCAAGAGTTGATCCATC | *Zm00001d047946* off-target site |
| OFT-JCR15 | CTGGAAAGACTTAGATGGCA |  |
| HF-TC1 | GGAGTGAGTACGGTGTGCCCTAATCGCCAGAGAAGTTC | Site TC1 |
| HR-TC1 | GAGTTGGATGCTGGATGGGACGAACAGACTGGCTGTGC |  |
| HF-TC2 | GGAGTGAGTACGGTGTGCCCTAATCGCCAGAGAAGTTC | Site TC2 |
| HR-TC2 | GAGTTGGATGCTGGATGGTAGCTTGTATCAAAAGATA |  |
| HF-TC3 | GGAGTGAGTACGGTGTGCCAGCGTCTTGACCAGCCTAT | Site TC3 |
| HR-TC3 | GAGTTGGATGCTGGATGGTCCACCAATGTTTGCAGGAA |  |
| HF-CC1/2 | GGAGTGAGTACGGTGTGCCTTCAATTGTGCTGTCTGGG | Site CC1/2 |
| HR-CC1/2 | GAGTTGGATGCTGGATGGCCTCGATAATGAAGATGATG |  |
| HF-CC3 | GGAGTGAGTACGGTGTGCCTGGCAGGTTCATAATGATG | Site CC3 |
| HR-CC3 | GAGTTGGATGCTGGATGGACTAAAGGTGAAAAGGTATGG |  |
| HF-GC1 | GGAGTGAGTACGGTGTGCTTGTGGAAGTCCAATTGTCA | Site GC1 |
| HR-GC1 | GAGTTGGATGCTGGATGGTTGCGGTCAATACATCTGGC |  |
| HF-GC2 | GGAGTGAGTACGGTGTGCCCTAAGCTCATAATGATGG | Site GC2 |
| HR-GC2 | GAGTTGGATGCTGGATGGATCGGAATTATAGGAGTGCC |  |
| HF-GC3 | GGAGTGAGTACGGTGTGCGACTGAAGAAGACTATGCTCGC | Site GC3 |
| HR-GC3 | GAGTTGGATGCTGGATGGAGCACTGGCAATAGCAGCAC |  |
| HF-AC1 | GGAGTGAGTACGGTGTGCGGACATGGGCTAATGATAC | Site AC1 |
| HR-AC1 | GAGTTGGATGCTGGATGGGAGTTGAACATTGGCGTAGT |  |
| HF-AC2 | GGAGTGAGTACGGTGTGCACAATGGAATGGCAGCAGCA | Site AC2 |
| HR-AC2 | GAGTTGGATGCTGGATGGGGCACCTCTACGAATTGGTC |  |
| HF-AC3 | GGAGTGAGTACGGTGTGCTGCTTATTCTAGGGCATATG | Site AC3 |
| HR-AC3 | GAGTTGGATGCTGGATGGCGCCCAAGGAGCTTGTTCAG |  |
| HF-AT1/2 | GGAGTGAGTACGGTGTGCCAGCGTCTTGACCAGCCTAT | Site ABE-AT1/2 |
| HR-AT1/2 | GAGTTGGATGCTGGATGGTCCACCAATGTTTGCAGGAA |  |
| HF-AT3 | GGAGTGAGTACGGTGTGCCCTAAGCTCATAATGATGG | Site ABE-AT3 |
| HR-AT3 | GAGTTGGATGCTGGATGGATCGGAATTATAGGAGTGCC |  |
| OFT-JCF1 | TAGCTCACGAGCTGCAACTA | *Zm00001d014294* off-target site |
| OFT-JCR1 | CTACAGCTATGACACCAACT |  |
| OFT-JCF2 | GGCCGTCATGCGTCTTGATG | *Zm00001d012641* off-target site |
| OFT-JCR2 | CGGCGGTTCTTAGCAGTATG |  |
| OFT-JCF3 | GTCAGTGTCGGCGTATGTGT | *Zm00001d040624* off-target site |
| OFT-JCR3 | CTAGCGACAGATACATGCTA |  |
| OFT-JCF4 | GGGTGGACAGATGGTCCAAT | *Zm00001d015699* off-target site |
| OFT-JCR4 | TCATCTGGCACCATCTCTTC |  |
| OFT-JCF5 | GTAAGACAATAGACAAGCTAAGG | *Zm00001d039630* off-target site |
| OFT-JCR5 | CATCTGGCACCATCTCTTCA |  |
| OFT-JCF6 | CCTTGTGACTTCGTTCCTTA | *Zm00001d047175* off-target site |
| OFT-JCR6 | CTCGAATACGCAGGAGAACT |  |
| OFT-JCF7 | CCTATAGGTGCATAGGACAG | *Zm00001d046039* off-target site |
| OFT-JCR7 | CTTGATTCAGATGCAGATTG |  |
| OFT-JCF8 | GTAGGTCACGCGTGCTAGAA | *Zm00001d048147* off-target site |
| OFT-JCR8 | TCACTGAGCTCATCAAGTTC |  |
| OFT-JCF9 | GAGTTAGCGATGGCCGGAGA | *Zm00001d047912* off-target site |
| OFT-JCR9 | ATCACAACATTGGCCTGGAG |  |
| OFT-JCF10 | CTCCAGCCTTACATTGATTG | *Zm00001d026249* off-target site |
| OFT-JCR10 | TCGACATTGTGTTCAGACCG |  |
| OFT-JCF11 | CCTATAGGTGCATAGGACAG | *Zm00001d046039* off-target site |
| OFT-JCR11 | CTTGATTCAGATGCAGATTG |  |
| OFT-JCF12 | CCAAGCGCTGGATCACCAAG | *Zm00001d023384* off-target site |
| OFT-JCR12 | TCACGGCTTGCCCTTGTGCT |  |
| OFT-JCF13 | CGTAAACTTGTGGTGGCTGA | *Zm00001d049210* off-target site |
| OFT-JCR13 | TGACTACGCTCCTGCATGAA |  |
| OFT-JCF14 | CCATGCTTCTATCATGGAAC | *Zm00001d043434* off-target site |
| OFT-JCR14 | GTCCCAACATAATCCTGTGG |  |
| OFT-JCF15 | TCACCAAGAGTTGATCCATC | *Zm00001d047946* off-target site |
| OFT-JCR15 | CTGGAAAGACTTAGATGGCA |  |

Table S6. The sequences of the gene fragments for evoAPOBEC1, evoCDA1, evoFERNY, RrA3F, TadA8.20, TadA8e, and the tGly-T1-esgRNA scaffold-HDV-tMet structure.

| Fragments | Nucleotide sequences (5’-3’) |
| --- | --- |
| evoAPOBEC1 | TCAAAGACTGGGCCTGTCGCCGTCGATCCAACCCTGCGCCGCCGGATTGAACCTCACGAGTTTGAAGTGTTCTTTGACCCTCGGGAGCTGAGAAAGGAGACATGCCTGCTGTACGAGATCAACTGGGGAGGCAGGCACTCCATCTGGAGGCACACCTCTCAGAACACAAATAAGCACGTCGAGGTGAACTTCATCGAGAAGTTTACCACAGAGCGGTACTTCTGCCCCAATACCAGATGTAGCATCACATGGTTTCTGAGCTGGTCCCCTTGCGGAGAGTGTAGCAGGGCCATCACCGAGTTCCTGTCCAGATATCCAAATGTGACACTGTTTATCTACATCGCCAGGCTGTATCACCTGGCAAACCCAAGGAATAGGCAGGGCCTGCGCGATCTGATCAGCTCCGGCGTGACCATCCAGATCATGACAGAGCAGGAGTCCGGCTACTGCTGGCACAACTTCGTGAATTATTCTCCTAGCAACGAGTCCCACTGGCCTAGGTATCCACACCTGTGGGTGCGCCTGTACGTGCTGGAGCTGTATTGCATCATCCTGGGCCTGCCCCCTTGTCTGAATATCCTGCGGAGAAAGCAGAGCCAGCTGACCTCCTTTACAATCGCCCTGCAGTCTTGTCACTATCAGAGGCTGCCACCCCACATCCTGTGGGCCACAGGCCTGAAG |
| evoCDA1 | ACCGACGCCGAGTACGTGCGGATTCACGAGAAGCTGGATATCTATACATTCAAGAAGCAGTTTAGCAACAATAAGAAGTCCGTGTCTCACAGATGCTACGTGCTGTTCGAGCTGAAGCGGAGAGGAGAGAGGCGCGCCTGTTTTTGGGGCTATGCCGTGAACAAGCCACAGTCTGGAACCGAGAGGGGAATCCACGCAGAGATCTTCAGCATCAGGAAGGTGGAGGAGTACCTGCGCGACAACCCCGGCCAGTTTACAATCAATTGGTATAGCTCCTGGAGCCCTTGCGCCGATTGTGCCGAGAAGATCCTGGAGTGGTACAACCAGGAGCTGAGGGGCAATGGCCACACCCTGAAGATCTGGGTGTGCAAGCTGTACTATGAGAAGAACGCCAGGAATCAGATCGGCCTGTGGAACCTGCGCGACAATGGCGTGGGCCTGAACGTGATGGTGTCCGAGCACTATCAGTGCTGTCGCAAGATCTTTATCCAGTCTAGCCACAATCAGCTGAACGAGAATCGGTGGCTGGAGAAGACACTGAAGAGAGCCGAGAAGCGGAGAAGCGAGCTGTCCATCATGTTTCAGGTGAAGATCCTGCACACCACAAAGTCTCCCGCCGTG |
| evoFERNY | TTTGAGAGGAACTACGACCCCCGCGAGCTGAGAAAGGAGACATACCTGCTGTATGAGATCAAGTGGGGCAAGTCCGGCAAGCTGTGGAGGCACTGGTGCCAGAACAATCGCACACAGCACGCCGAGGTGTACTTCCTGGAGAACATCTTTAATGCCCGGAGATTCAATCCATCTACCCACTGTAGCATCACATGGTATCTGAGCTGGTCCCCCTGCGCCGAGTGTTCTCAGAAGATCGTGGATTTCCTGAAGGAGCACCCTAACGTGAATCTGGAGATCTATGTGGCCCGGCTGTACTATCCAGAGAACGAGAGGAATAGGCAGGGCCTGCGGGATCTGGTGAATTCCGGCGTGACCATCAGAATCATGGACCTGCCAGATTACAACTATTGCTGGAAGACCTTCGTGAGCGATCAGGGAGGCGACGAGGATTACTGGCCAGGACACTTCGCCCCTTGGATCAAGCAGTATAGCCTGAAGCTG |
| RrA3F | AAGCCCCAGATCAGGGACCACCGCCCCAATCCTATGGAGGCCATGTACCCTCACATCTTCTATTTTCACTTCGAGAACCTGGAGAAGGCCTACGGCCGGAATGAGACCTGGCTGTGCTTTACAGTGGAGATCATCAAGCAGTATCTGCCAGTGCCCTGGAAGAAGGGCGTGTTCCGGAACCAGGTGGACCCAGAGACCCACTGCCACGCCGAGAAGTGTTTTCTGTCCTGGTTCTGTAACAATACACTGTCTCCCAAGAAGAATTACCAGGTGACATGGTATACAAGCTGGTCCCCTTGCCCAGAGTGTGCAGGAGAGGTGGCAGAGTTTCTGGCAGAGCACAGCAACGTGAAGCTGACCATCTACACAGCCCGGCTGTACTATTTCTGGGACACCGATTATCAGGAGGGCCTGAGATCTCTGAGCGAGGAGGGCGCCTCCGTGGAGATCATGGACTACGAGGATTTTCAGTATTGCTGGGAGAACTTCGTGTACGACGATGGCGAGCCATTCAAGAGGTGGAAGGGCCTGAAGTATAATTTCCAGTCTCTGACACGGAGACTGCGCGAGATCCTGCAG |
| TadA8.20 | TCTGATGAGGTGGAGTTTTCCCACGAGTACTGGATGAGACATGCCCTGACCCTGGCCAAGAGGGCACGCGATGAGAGGGAGGTGCCTGTGGGAGCCGTGCTGGTGCTGAACAATAGAGTGATCGGCGAGGGCTGGAACAGAGCCATCGGCCTGCACGACCCAACAGCCCATGCCGAAATTATGGCCCTGAGACAGGGCGGCCTGGTCATGCAGAACTACAGACTGTACGACGCCACCCTGTACTCCACATTCGAGCCTTGCGTGATGTGCGCCGGCGCCATGATCCACTCTAGGATCGGCCGCGTGGTGTTTGGCTGGAGGAACGCCAAAACCGGCGCCGCAGGCTCCCTGATGGACGTGCTGCACCACCCCGGCATGAATCACCGCGTCGAAATTACCGAGGGAATCCTGGCAGATGAATGTGCCGCCCTGCTGTGCAGATTCTTCCGGATGCCTAGAAGAGTGTTCAATGCTCAGAAGAAGGCCCAGAGCTCCATCAAC |
| TadA8e | TCTGAGGTGGAGTTTTCCCACGAGTACTGGATGAGACATGCCCTGACCCTGGCCAAGAGGGCACGCGATGAGAGGGAGGTGCCTGTGGGAGCCGTGCTGGTGCTGAACAATAGAGTGATCGGCGAGGGCTGGAACAGAGCCATCGGCCTGCACGACCCAACAGCCCATGCCGAAATTATGGCCCTGAGACAGGGCGGCCTGGTCATGCAGAACTACAGACTGATTGACGCCACCCTGTACGTGACATTCGAGCCTTGCGTGATGTGCGCCGGCGCCATGATCCACTCTAGGATCGGCCGCGTGGTGTTTGGCTGGAGGAACTCCAAACGCGGCGCCGCAGGCTCCCTGATGAACGTGCTGAACTACCCCGGCATGAATCACCGCGTCGAAATTACCGAGGGAATCCTGGCAGATGAATGTGCCGCCCTGCTGTGCGATTTCTACCGGATGCCTAGACAGGTGTTCAATGCTCAGAAGAAGGCCCAGAGCTCCATCAAC |
| tGly-T1-esgRNA scaffold-HDV-tMet | GCACCAGTGGTCTAGTGGTAGAATAGTACCCTGCCACGGTACAGACCCGGGTTCGATTCCCGGCTGGTGCAGtcctcttgtttcaggaagaGTTTCAGAGCTATGCTGGAAACAGCATAGCAAGTTGAAATAAGGCTAGTCCGTTATCAACTTGAAAAAGTGGCACCGAGTCGGTGCGGCCGGCATGGTCCCAGCCTCCTCGCTGGCGCCGGCTGGGCAACATGCTTCGGCATGGCGAATGGGACAACAACAAATCAGAGTGGCGCAGCGGAAGCGTGGTGGGCCCATAACCCACAGGTCCCAGGATCGAAACCTGGCTCTGATAgacctctggtttccggtaggGTTTCAGAGCTATGCTGGAAACAGCATAGCAAGTTGAAATAAGGCTAGTCCGTTATCAACTTGAAAAAGTGGCACCGAGTCGGTGCGGCCGGCATGGTCCCAGCCTCCTCGCTGGCGCCGGCTGGGCAACATGCTTCGGCATGGCGAATGGGACAACAACAAGCACCAGTGGTCTAGTGGTAGAATAGTACCCTGCCACGGTACAGACCCGGGTTCGATTCCCGGCTGGTGCAggcctcttgtttcaggaaggGTTTCAGAGCTATGCTGGAAACAGCATAGCAAGTTGAAATAAGGCTAGTCCGTTATCAACTTGAAAAAGTGGCACCGAGTCGGTGCGGCCGGCATGGTCCCAGCCTCCTCGCTGGCGCCGGCTGGGCAACATGCTTCGGCATGGCGAATGGGAC |
